# *t*-Butyl and Trimethylsilyl Substituents in Nickel Allyl Complexes: Similar but Not the Same

**DOI:** 10.1021/acsorginorgau.4c00044

**Published:** 2024-09-17

**Authors:** Henry P. DeGroot, Isaiah R. Speight, William W. Brennessel, Timothy P. Hanusa

**Affiliations:** † Department of Chemistry, Vanderbilt University, PO Box 1822 Nashville, Tennessee 37235, United States; ‡ X-ray Crystallographic Facility, Department of Chemistry, University of Rochester, Rochester, New York 14627, United States

**Keywords:** allyl, nickel, mechanochemistry, steric bulk, *t*-butyl, trimethylsilyl

## Abstract

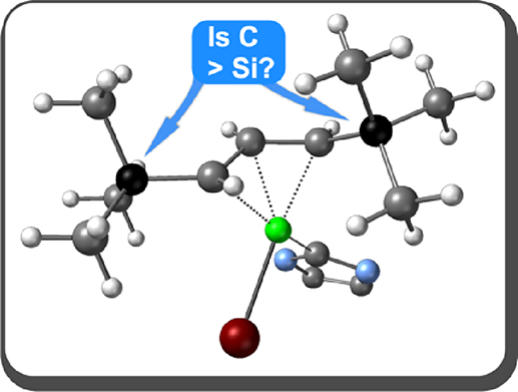

Metal complexes with *t*-Bu-substituted allyl ligands are relatively rare, especially compared to their conceptually similar trimethylsilyl-substituted analogs. The scarcity partially stems from the few general synthetic entry points for the *t*-Bu versions. This situation was studied through a modified synthesis for the allyl ligand itself and by forming several mono(allyl)nickel derivatives. After 2,2,6,6-tetramethyl-4-hepten-3-one was converted to the related 5-bromo-2,2,6,6-tetramethylhept-3-ene (A^2t^Br), a mixture of Ni(COD)_2_ and A^2t^Br in the presence of a neutral donor ligand such as MeCN was found to produce the dark red dimeric π-allyl complex [{A^2t^NiBr}_2_]. Both NMR and X-ray crystallographic data confirmed that the *t*-Bu substituents are in a *syn*, *syn*-conformation, like that in the previously described [{A′NiBr}_2_] (A′ = 1,3-(TMS)_2_C_3_H_3_) complex. [{A^2t^NiBr}_2_] will form adducts with neutral donors such as PPh_3_ and IMes (IMes = 1,3-dimesitylimidazol-2-ylidene), but the resulting [A^2t^Ni(PPh_3_)Br] complex is not as stable as its trimethylsilyl analog. The [A^2t^Ni(IMes)Br] complex crystallizes from hexanes as a monomer, with an η^3^-coordinated [A^2t^] ligand, and in contrast to the starting arrangement in [{A^2t^NiBr}_2_], the *t*-Bu groups on the A^2t^ ligand are in a *syn*, *anti-*relationship. This structure is paralleled in the trimethylsilyl analog [A′Ni(IMes)Br]. DFT calculations were used to compare the structures of *t*-Bu- and related trimethylsilyl-substituted complexes.

## Introduction

Transition metal allyl complexes are staple participants in many organic transformations, including cross-coupling reactions and catalytic polymerizations.^1−8^ As they provide useful steric bulk and kinetic stability, silyl-substituted allyl ligands,^9,10^ particularly those with trimethylsilyl (TMS) groups, are often valuable in these contexts.^11−15^ Nevertheless, silyl groups are typically labile in acidic media or with fluoride-containing precursors, which can restrict their range of applications.^16−18^ There may also be direct electronic effects of trimethylsilyl substituents that contribute to their unsuitability in specific cases. For example, TMS groups commonly function as electron donors,^19,20^ but they can also exhibit the β -silicon effect, in which Si–C hyperconjugation serves to weaken metal–ligand bonds in delocalized systems such as allyls and indenyls.^21−24^

Although it is not a drop-in replacement for TMS, the *t*-butyl group is, of course, conceptually related,^25^ and substituted allyl ligands such as [1-(*t*-Bu)C_3_H_4_] (= A^1t^) and [1,3-(*t*-Bu)_2_C_3_H_3_] (= A^2t^) have been incorporated into metal complexes.^26−29^ However, *t*-Bu-substituted allyl metal complexes remain a relatively rare and understudied class of compounds compared to their silyl analogs. Apart from difficulties synthesizing such complexes (see below), some display instability ascribed to the steric bulk of the *t*-Bu substituents. For example, both [TpMo(CO)_2_A^1t^] and [TpMo(CO)_2_A^2t^] (Tp = trispyrazolylborate) decompose rapidly as solids or on standing in solution.^27^ In contrast, the analogous complexes with methyl or phenyl substituents on the allyl ligands are stable in solution and only slowly decompose on exposure to air. Nevertheless, the successful application of, for example, the related 1-(*t*-Bu)-indenyl ligand to palladium catalysis suggests that an improved understanding of *t*-Bu-substituted allyls and their metal complexes could have useful consequences.^7^

**Figure 1:**
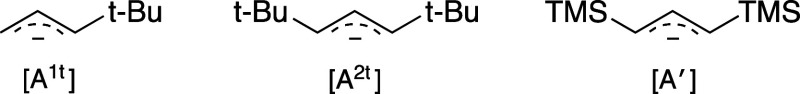
Abbreviations for substituted allyl ligands mentioned in the text.

The synthesis of *t*-Bu-substituted allyl complexes is typically different from the routes used for TMS analogs. For the latter, the alkali metal salts of the [1-(TMS)C_3_H_4_] and [1,3-(TMS)_2_C_3_H_3_] (= [A′]) anions are readily prepared (Figure 1), and metathesis with main-group and transition metal halides is a broadly effective method.^9,10^ Although NMR characterization of [(tmeda)Li][A^2t^] has been reported,^26^ to our knowledge no metathesis reactions have been described using *t*-Bu-substituted allyl salts. However, various other methods have been used in synthesizing *t*-Bu-substituted allyl complexes, usually from a neutral substituted precursor or by assembling the ligand *in situ*. For example, [{(1-*t*-Bu)(2-Me)C_3_H_3_]Pd(μ-Cl)}_2_] was formed from [PdCl_4_]^2–^ and 2,4,4-trimethyl-2-pentene,^30^ and the osmium-coordinated allyl ligand in the trinuclear cluster [Os_3_(CO)_8_(μ-CO)(μ–γ-C_5_H_3_O_2_)A^1t^] was generated during the photochemical reaction of [Os_3_(CO)_10_(μ-H)(μ–γ-C_5_H_3_O_2_)] with *t*-BuC≡CMe.^28^ The trispyrazolylborate derivative [TpMo(CO)_2_A^1t^] noted above was synthesized from the reaction of 3-acetoxy-4,4-dimethyl-1-pentene, [(DMF)_3_Mo(CO)_3_], and K[Tp].^27^

**Figure 2:**
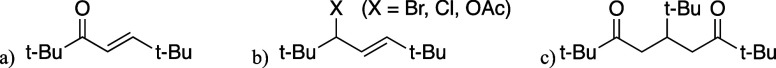
(a) 2,2,6,6-tetramethyl-4-hepten-3-one (**1**); (b) 5-(bromo, chloro, acetoxy)-2,2,6,6-tetramethylhept-3-ene; (c) 5-(*t*-Bu)-2,2,8,8-tetramethylnonane-3,7-dione (**2**).

Apart from the *in situ* pathways,^28^ routes to complexes containing the [A^2t^] ligand typically involve the use of 2,2,6,6-tetramethyl-4-hepten-3-one (**1**, Figure 2a), which is converted into a halo or acetoxy derivative (Figure 2b) before being attached to a metal center. The ketone, in turn, is commonly obtained through Aldol condensation of pinacolone and pivaldehyde to form the propene backbone (Table 1). Reported yields through this “classic” route are low-to-moderate (23–37% with NaOEt as the base),^31,32^ although higher-yielding routes are available from a hydrostannation/Stille coupling sequence.^33^ However, given the ease of the single-step condensation method, we were interested in refining its reaction conditions. In the course of this work, an improved route to the known diketone (**2**, Figure 2c) was discovered.

**Table 1:**
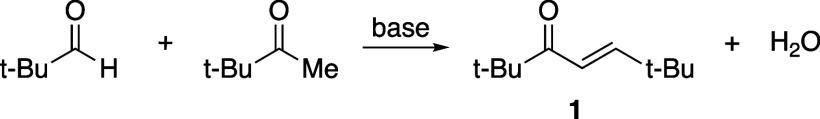
Comparative Reaction Conditions for the Synthesis of Ketone **1**

**entry**	**conditions**	**yield %**	**ref**
1	NaOH/EtOH, 48 h, RT	23–37	(31,32)
2	Li[N(TMS)_2_]/THF, −78 °C → RT (overnight)	69	this work
3	NaOH, mixer mill, 30 min, 30 Hz	52	this work

Among the first allyl complexes synthesized were those containing nickel,^34^ and nickel derivatives are still critical in the development of high-performance olefin polymerization catalysts.^35−38^ To examine the effect of *t*-butyl substitution on the allyl ligand, several nickel allyl complexes containing the [A^2t^] ligand were prepared from the bromo alkene (Figure 2b, with X = Br), which allow direct comparisons with TMS-substituted derivatives.

## Results

### Synthesis of 2,2,6,6-Tetramethyl-4-hepten-3-one (**1**) and the corresponding dione (**2**) and pivalate (**3**)

Aldol condensation of pinacolone and pivaldehyde commonly employs ethanolic NaOH as a base (Table 1, entry 1).^31,32,39^ The yield of **1** can be raised to 69%, effectively doubling literature yields, by using Li[N(TMS)_2_] in toluene at low temperature (Table 1, entry 2). The reaction is readily scalable to the 20 g level.

**Figure 3:**
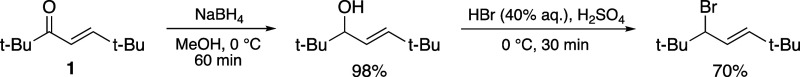
Conversion of **1** into the corresponding alcohol and bromide.^42^

Aldol reactions with no added solvents can offer efficient conversion rates with the appropriate substrates,^40^ and a mechanochemical route has been described using a solution-prepared Li pinacolone enolate as a reagent.^41^ We find an even simpler mechanochemical preparation is possible by grinding pinacolone, pivaldehyde, and NaOH for 30 min in a mixer mill. When followed by workup with aqueous HCl, the reaction provides **1** in 52% yield (Table 1, entry 3). Subsequent sodium borohydride reduction of **1** produces the corresponding alcohol^42^ (Figure 3); this procedure has been performed on a multigram scale (up to 8 g). The alcohol is converted to the bromo species (A^2t^Br) with HBr,^42^ or to the chloro analogue (A^2t^Cl) by reaction with thionyl chloride.^26^

**Figure 4:**
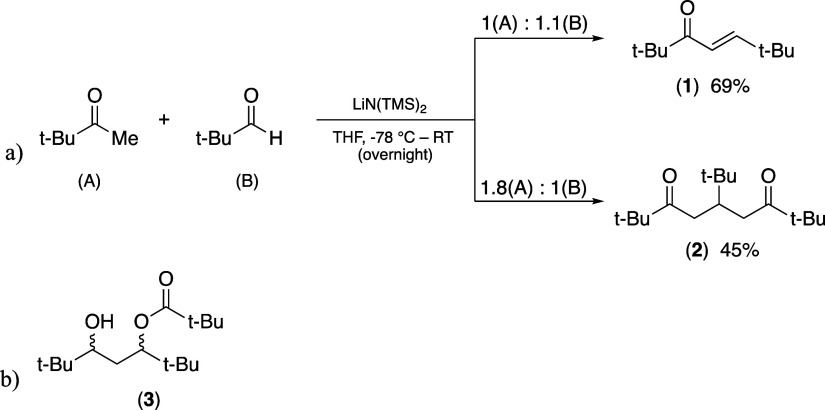
Molar equivalence influence on the aldol condensation reaction; (a) **2** is formed when pinacolone is in excess; (b) structure of 5-hydroxy-2,2,6,6-tetramethylheptan-3-yl pivalate (**3**), formed in minor amounts in a racemic mixture when pivaldehyde is in excess.

During optimization experiments of the aldol condensation for **1**, the highly crystalline double addition product species 2,2,8,8-tetramethyl-5-(*t*-butyl)-3,7-nonanedione (**2**)^43−45^ was isolated in moderate yield when pinacolone was in excess (Figure 4a). Other routes have previously been described for **2**, such as the reaction of pinacolone with **1**, which provides a slightly greater yield (59%) but requires the prior preparation of **1**.^45^ The diketone has been used in the preparation of thiopyrylium salts.^44,46^

**Figure 5:**
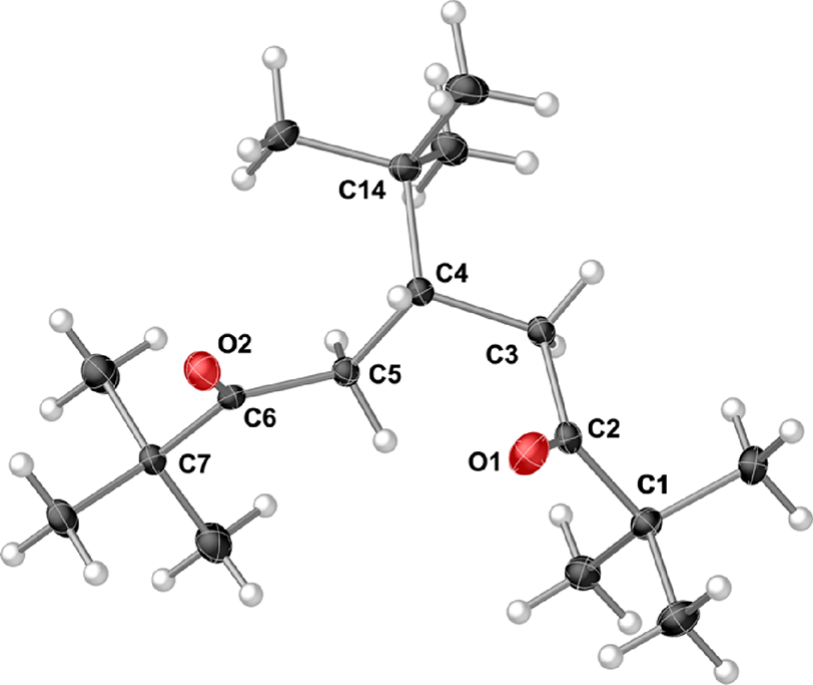
Thermal ellipsoid plot (50% level) of 5-(*tert*-butyl)-2,2,8,8-tetramethylnonane-3,7-dione (**2**). Selected bond distances (Å) and angles (deg): O1–C2, 1.2159(12); O2–C6, 1.2142(12); C1–C2, 1.5340(13); C2–C3, 1.5225(13); C3–C4, 1.5392(13); C4–C5, 1.5392(13); C4–C14, 1.5557(13); C5–C6 1.5150(12); C6–C7, 1.5393(13); C2–C3–C4–C5, −73.45.

The crystal structure of **2** shows that the carbonyls are oriented *cis* to each other (Figure 5). As there are no close intramolecular contacts, it seems that other configurations might be similarly favorable. DFT calculations (B3PW91-D3BJ/def2-TZVP) comparing the *cis* and an alternative *trans* configuration indicates that the two versions are effectively indistinguishable in energy (Δ*G*° = 0.50 kJ mol^–1^ in favor of the *trans* form). Packing forces are presumably responsible for the observed solid-state arrangement.

When pivaldehyde is used in excess, 5-hydroxy-2,2,6,6-tetramethylheptan-3-yl pivalate (**3**) is frequently formed as a minor byproduct (Figure 4b). To our knowledge, this compound has not been previously reported. It likely forms via deprotonation of the initial condensation product to form an enolate, followed by an O-centered attack on another molecule of pivaldehyde. The observance of this product and the complete absence of the product of C-centered attack likely implicates steric influence in directing reactivity. Only one set of peaks is observed in the proton NMR spectrum; taken in conjunction with the structure obtained from a few isolated crystals of the compound (see Figure S1), this suggests that only the *anti*-enantiomer is formed.

### Synthesis of *t*-Butyl-Substituted (Allyl)nickel Bromides

**Figure 6:**
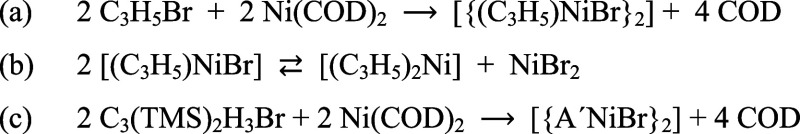
Reactions of (allyl)nickel bromides.

(Allyl)nickel bromide compounds were prepared to allow comparisons between complexes of the parent allyl [C_3_H_5_], the TMS-substituted [A′], and the *t*-Bu derivative [A^2t^] ligands. In general, (allyl)nickel bromides are red to red-purple, air- and moisture-sensitive compounds that can be prepared in several ways,^47,48^ the most relevant of which involves oxidative addition between an allyl bromide and a source of Ni(0), such as bis(cyclooctadiene)nickel (e.g., Figure 6a).^47^ The bromide complex with the parent allyl is a dimer in aromatic hydrocarbons (i.e., [{(C_3_H_5_)NiBr}_2_]), but Schlenk equilibrium is established if the compound is dissolved in a coordinating donor solvent such as DMF or HMPA (Figure 6b).^49^

The oxidative addition method of (allyl)nickel halide preparation is also successful with TMS-substituted allyls; for example, the bis(trimethylsilyl)allyl bromide 1,3-(TMS)_2_C_3_H_3_Br reacts with Ni(COD)_2_ in toluene to generate the substituted (allyl)nickel bromide (Figure 6c).^13^ In contrast to [{(C_3_H_5_)NiBr}_2_], [{A′NiBr}_2_] is stable to rearrangement in solution, i.e., there is no NMR evidence for the formation of [NiA′_2_] via Schlenk rearrangement in THF.

Given this precedent, it was somewhat surprising that the mixture of A^2t^Br and Ni(COD)_2_ in THF did not react to form a nickel complex (Table 2, entry 1). The allyl halide instead initiated the decomposition of Ni(COD)_2_ to nickel metal, with a black solid visible within 5 min of addition. Workup of the remaining solution revealed the presence only of free COD and unreacted allyl halide. Modifying the reaction conditions, e.g., with UV irradiation or by conducting the reaction at reflux (66 °C), did not affect the outcome of the reaction. As a test, the addition of two extra equivalents of COD to the reaction mixture did not substantially retard the decomposition, suggesting that the displacement of COD by A^2t^Br was not a rate-limiting step. If the allylic chloride A^2t^Cl is used in place of the bromide, the rate of decomposition is slower, but nickel metal is still deposited within 30 min.

**Table 2:**
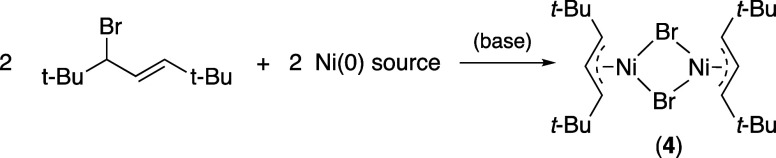
Comparative Reaction Conditions for the Synthesis of **4** ([{A^2t^NiBr}_2_])

**entry**	**nickel source**	**base**	**conditions**	**yield %**
1^a^	Ni(COD)_2_	none	THF (RT or reflux), 5 min	0
2	Ni(CO)_4_	none	THF	0
3	[Ni(CO)_2_(PPh_3_)_2_]	none	toluene (70 °C)	trace
4	Ni(COD)_2_	PPh_3_, 1 equiv.	THF	40–60
5	Ni(COD)_2_	IMes, 1 equiv.	THF	40–60
6	Ni(COD)_2_	MeCN, 1 equiv.	THF	40–60
7	Ni(COD)_2_	MeCN, 10 equiv.	THF	70
8	Ni(COD)_2_	MeCN	MeCN (neat)	37

a No product was observed under UV radiation.

Modifying the coordination environment of the nickel was eventually found to be critical to generating an allyl complex. Although a test using Ni(CO)_4_ and A^2t^Br was not successful (entry 2), a few crystals of the desired bromide-bridged nickel complex, [{A^2t^NiBr}_2_] (**4**), were obtained when [Ni(CO)_2_(PPh_3_)_2_] was used as the Ni(0) source in toluene solution (entry 3). However, only trace quantities could be obtained by this route, with most of the recovered material being unreacted A^2t^Br.

**Figure 7:**
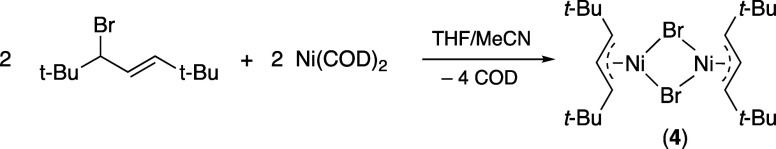
Formation of the (allyl)nickel bromide [{A^2t^NiBr}_2_]. Other neutral donors that can be used in place of MeCN include PPh_3_ and IMes.

A series of optimization experiments was conducted that ultimately led to useful quantities of **4** (Table 2). A substantial improvement came through the discovery that the original mixture of Ni(COD)_2_ and A^2t^Br in THF yielded **4** when one equiv of an additional neutral donor ligand (L = PPh_3_, IMes, or MeCN) was added to the reaction (entries 4–6; Figure 7), increasing yields to 40–60%. Although encouraging, the still-moderate yields of **4** and the absence of the ancillary ligands in the product (which would be expected to form adducts, see below) suggested that some nickel was prematurely captured, thereby being unavailable for oxidative addition. In all cases, hexane-insoluble material was present in the reaction mixtures, which may have comprised ligand adducts of partially oxidized nickel. Evidence for this was obtained from colorless crystals isolated as a byproduct when PPh_3_ was used as the auxiliary base. Their unit cell matched that for [Ni(PPh_3_)_3_Br],^50^ confirming the presence of incompletely oxidized nickel. An analogous Ni(I) species is known with IMes (i.e., [Ni(IMes)_2_Br]),^51^ which could serve as a nickel trap. Incomplete oxidation is further evidenced by the consistent presence of unreacted A^2t^Br and the coupled hexadiene {A^2t^}_2_ in the crude product mixture.

Not all neutral donor ligands are effective additives, however. For example, adding pyridine to a mixture of Ni(COD)_2_ and A^2t^Br in THF did not cause the swift color change associated with oxidative addition. Pyridine also seemed to suppress the decomposition of Ni(COD)_2_, as no metallic nickel was observed after 10 min of stirring. Subsequent addition of acetonitrile initiated the expected oxidative addition reaction, however.

Acetonitrile was eventually found to be the superior additive, and an excess of the ligand could be used without irreversible incorporation into the product. When 10 equiv of MeCN were used in the reaction, [{A^2t^NiBr}_2_] could be recovered in an average isolated yield of 70% (Table 2, entry 7). Using MeCN as the sole reaction solvent led to a yield of only half that of a MeCN/THF mixture (entry 8), indicating that a mixed-solvent system is critical to an optimal reaction outcome.

Isolated **4** is a chalky red solid that appears stable for months under nitrogen in the solid state or in hydrocarbon solution. It is slightly soluble in hexanes, soluble in aromatic hydrocarbons, and highly soluble in polar organic solvents. As with [{A′NiBr}_2_], an NMR spectrum taken in a Lewis basic solvent (DMSO-*d*_6_) shows no evidence for the operation of Schlenk equilibrium.

Even though both PPh_3_ and IMes were suboptimal additives, their use did reveal another example of the distinctive reactivity of the A^2t^Br/Ni(0) system. It is commonly found that oxidative addition of an allyl halide to nickel in the presence of one equivalent of phosphine or NHC yields adduct complexes [(allyl)Ni(L)X],^52−57^ but even when a full equivalent of either PPh_3_ and IMes was added to the mixture of Ni(COD)_2_ and BrA^2t^, unligated **4** was the dominant product. In the case of IMes, a corresponding adduct was completely absent, while in the case of PPh_3_, an adduct was inconsistently present as a minor product (10–25%) when more than 0.5 equiv. was used. The extent to which this new complex resists additional ligand binding will be discussed below. Interestingly, the inclusion of a donor ligand did not lead to a successful reaction when either [Ni(CO)_2_(PPh_3_)_2_] or Ni(CO)_4_ was used in place of Ni(COD)_2_ as a nickel(0) source. These results suggest that the clean oxidative addition of A^2t^Br to nickel requires specifically optimized conditions and reagents.

**Figure 8:**
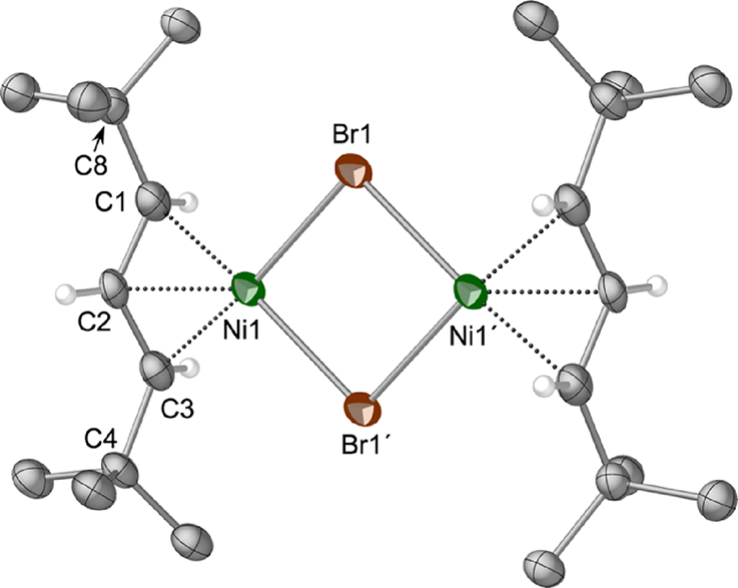
Thermal ellipsoid plot (50% level) of the structure of **4**; for clarity, hydrogen atoms have been removed from the *t*-Bu groups, and the rest have been given arbitrary radii. Selected bond distances (Å) and angles (deg): Ni1–Br(1), 2.3690(14); Ni1–Br(1)′, 2.3636(15); Ni(1)–C(1), 2.041(8); Ni(1)–C(2), 1.975(7); Ni(1)–C(3), 2.053(8); C(1)–C(2), 1.396(11); C(2)–C(3), 1.407(11); C(3)–C(4), 1.518(10); C(1)–C(8), 1.527(11); C(1)–C(2)–C(3), 118.7(7); C(3)–C(2)–C(1)–C(8), 179.85; C(1)–C(2)–C(3)–C(4), −179.90.

The allyl complex **4** crystallizes from hexanes as a bromide-bridged dimer, joining a family of bridged π-allyl nickel species.^13,58−64^ The molecule lies on a crystallographic inversion center, thus only one-half of the molecule is unique (Figure 8).

The Ni–C bond lengths range from 1.975(7) to 2.053(8) Å, which are within 0.01 Å of those in [{A′NiBr}_2_].^13^ Although not required to be so by symmetry, the Ni(1)–Br(1) and Ni(1)–Br(1)′ bond lengths are identical within 2σ (2.366(2) Å, ave.), and also track with the Ni–Br bonds in the TMS-substituted analog (2.362, 2.365 Å). The Ni(1)···Ni(1)′ separation is 3.39(1) Å, too long to represent any significant interaction (cf. the 2.49 Å distance in nickel metal^65^). The angle between the C_3_ plane and (NiBr)_2_ plane is 116.5°.

The carbon atoms C(4) and C(8) lie almost exactly in the C_3_ plane (0.0005 and 0.004 Å displacements, respectively). This configuration has been observed before in other bridged allyl dimers of nickel, such as {(1,3-Me_2_C_3_H_3_)Ni(μ-Me)}_2_,^62^ {[1-Me-3-(OSiMe_3_)C_3_H_3_]NiCl}_2_,^60^ and [{NiBrA′}_2_].^13^

**Figure 9:**
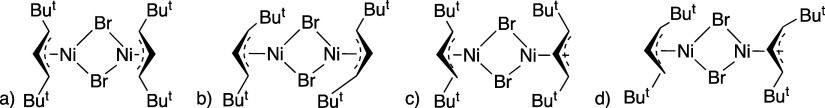
Possible stereoisomers of **4**: (a) staggered, with *syn*, *syn t*-Bu groups (as observed in the solid state); (b) staggered, with *syn*, *anti t*-Bu groups; (c) eclipsed, with *syn*, *syn t*-Bu groups; (d) eclipsed, with *syn*, *anti t*-Bu groups.

As with the ^1^H NMR spectrum of [{A′NiBr}_2_],^13,66^ the ^1^H NMR spectrum of **4** indicates that the *t*-Bu groups are equivalent in the primary species, and hence in a *syn*, *syn* arrangement, which is also that found in the solid state. Unlike its trimethylsilylated analog, minor amounts of its *syn*, *anti*-isomers do not appear to be present. Although two stereoisomers (with eclipsed or staggered allyl ligands) of the bis(*syn, syn*) complex are conceivable (Figure 9), only one set of signals is observed, indicating that either exclusively one form is present or that they interchange rapidly in solution. To probe this, a computational investigation of the possible stereoisomers of [{A^2t^NiBr}]_2_ was undertaken. In accordance with the observed NMR features, complexes with *syn*, *anti*-configurations were consistently found to have higher energy than the corresponding *syn*, *syn* complexes. For example, the eclipsed *syn,syn*/*syn,anti* configuration was found to be approximately 12 kJ mol^–1^ higher in energy (Δ*G*°) than the eclipsed *syn,syn*/*syn,syn* configuration, and with both allyl ligands in the *syn*,*anti* arrangement, the energy was from 20 to 26 kJ mol^–1^ higher in energy (see the SI for more details). In addition, the eclipsed conformations were consistently more stable than the corresponding staggered conformations, although the differences were less (ca. 6–10 kJ mol^–1^). This finding was unexpected, as the solid-state structure of the molecule (see below) exclusively contains staggered allyls. The free energy difference between the most stable eclipsed and staggered isomers was found to be <10 kJ mol^–1^, however, which could plausibly be the result of differences in crystal packing.^67^

### Ligand Adducts of [A^2t^NiBr]

**Figure 10:**
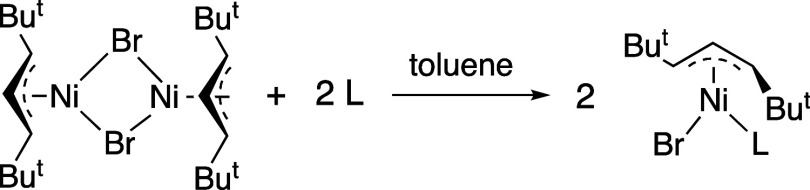
Formation of ligand adducts of [A^2t^NiBr]; L = PPh_3_, IMes.

As noted above, the reaction of bridged allyl nickel halide compounds with monodentate neutral donor ligands is well documented,^52−57^ generating monomeric adduct complexes. Accordingly, the reaction of **4** with 2 equiv of ligand (L = PPh_3_, IMes; 1 equiv. per metal center) generates new complexes consistent with such adducts, as evidenced with ^1^H NMR spectra (Figure 10).

In the case of triphenylphosphine, the reaction does not proceed to completion, forming an approximately 2:1 mixture of a monomeric adduct to the unreacted dimeric complex. Although the ^1^H NMR signal for the center allyl proton of the phosphine adduct is sharp, the terminal proton signals are substantially broadened; free and bound triphenylphosphine peaks are observed and are also broadened. Additionally, only one peak is present in the ^31^P NMR spectrum in C_6_D_6_ (δ 23.7), well downfield of free triphenylphosphine (δ −4.7),^68^ suggesting that phosphine binding is reversible, and that exchange is slow on the ^1^H NMR time scale but fast on the ^31^P time scale. Variable temperature (VT) NMR experiments showed that the ratio of adducted to unadducted complex consistently increased as temperature decreased (see SI for details). This process reversed upon warming back to room temperature. The allyl protons’ chemical shifts and coupling constants suggest a *syn*, *anti*-orientation. The mixture solidifies as a hard paste, and all attempts to crystallize the phosphine adduct have been unsuccessful. The adduct degrades over time, slowly in solution and more quickly in the solid state; the rate of solid-state degradation was found to vary over a period of weeks to months. The degradation process was found to yield the coupled hexadiene {A^2t^}_2_ while the [{A^2t^NiBr}_2_] appears unaffected. This observation, along with the lack of visible metallic nickel, suggests that triphenylphosphine remains bound to nickel after it is reduced and that there may be a complex mechanism for its decomposition.

The NMR ratio of [A^2t^Ni(PPh_3_)Br] to [{A^2t^NiBr}_2_] at various temperatures (298–253 K) was used to calculate the free energy change associated with adduct formation ([{A^2t^NiBr}_2_] + 2 PPh_3_ → 2[A^2t^Ni(PPh_3_)Br]; see the SI). At 298 K, Δ*G*° is only −1.7 kJ mol^–1^, which is consistent with the easily reversible binding of PPh_3_ to the nickel allyl complex. This number should be taken as an approximation, as a third species begins to be visible at low temperatures, likely an intermediate for PPh_3_ exchange (see Figure S9). A probable candidate for this species is [A^2t^Ni(PPh_3_)_2_Br], whose analogs are well documented,^69−71^ but a monomeric unit of [A^2t^NiBr] is also conceivable. As this species has not been identified, an exact equilibrium constant cannot be calculated. However, only minor amounts of this compound are present; at the minimum temperature observed, considering the new species to be either of the above complexes resulted in a maximum of 25% difference in the calculated Δ*G*° value. Therefore, the calculated values are likely reasonably accurate, especially at higher temperatures.

For comparison, PPh_3_ was also allowed to react with the trimethylsilyl analogue [{A′NiBr}_2_]. This reaction also produced a dark red paste that, like the result with [{A^2t^NiBr}_2_], resisted crystallization, but ^1^H NMR analysis revealed several differences from the reaction involving **4**. Most notable is the absence of any unreacted bimetallic complex; instead, only one species consistent with [A′Ni(PPh_3_)Br] is present in the spectrum. When a slight excess of free triphenylphosphine is present, it is observed in the ^31^P spectrum as a distinct peak at its expected chemical shift.^72^ Together, this suggests that the adduct formation reaction goes to completion and that any exchange processes are slow on the NMR time scale. A final difference from the *t*-Bu analogue is that the [A′] adduct appears stable in the solid state under N_2_, showing no apparent decomposition over a month. Analysis of the ^1^H–^1^H, ^1^H–^31^P, and ^13^C–^31^P coupling constants allowed unambiguous determination of the geometry of the molecule: the allyl is present in a *syn*, *anti*-configuration with the phosphine bound *trans* to the *syn*-TMS_3_ group, similar to that in [A^2t^Ni(PPh_3_)Br].

In the case of IMes, the new compound **5** displays sharp [A^2t^] allyl resonances at room temperature, and there is no evidence for free IMes. The molecule is sparingly soluble in hexanes, but highly soluble in aromatic and polar organic solvents. The mixture shows minimal degradation over months when stored in the solid state but appears to degrade in weeks when dissolved in hexanes. The compound has a *syn*, *anti*-configuration in solution, as evidenced by inequivalent *t*-butyl groups and a large difference between the two allylic coupling constants (*J* = 8.3, 14.6 Hz). The two imidazole methylene groups are equivalent, which implies that rotation around the carbene-Ni bond is fast on the NMR time scale. On the other hand, the *ortho* methyl groups and *meta* protons of the mesityl rings are both split into two separate signals, indicating that rotation about the N-Mes bond is slow. These NMR features are consistent with the reported NMR dynamics of an analogous complex featuring an unsubstituted allyl ligand and suggest that IMes exchange does not occur.^73^ A small amount of a second species is also consistently present whose NMR features are consistent with a *syn, syn* isomer of the complex. A computational investigation suggests that such a species is plausible (Δ*G*° = +5.2 kJ mol^–1^ relative to the *syn, anti*-isomer; see the SI).

**Figure 11:**
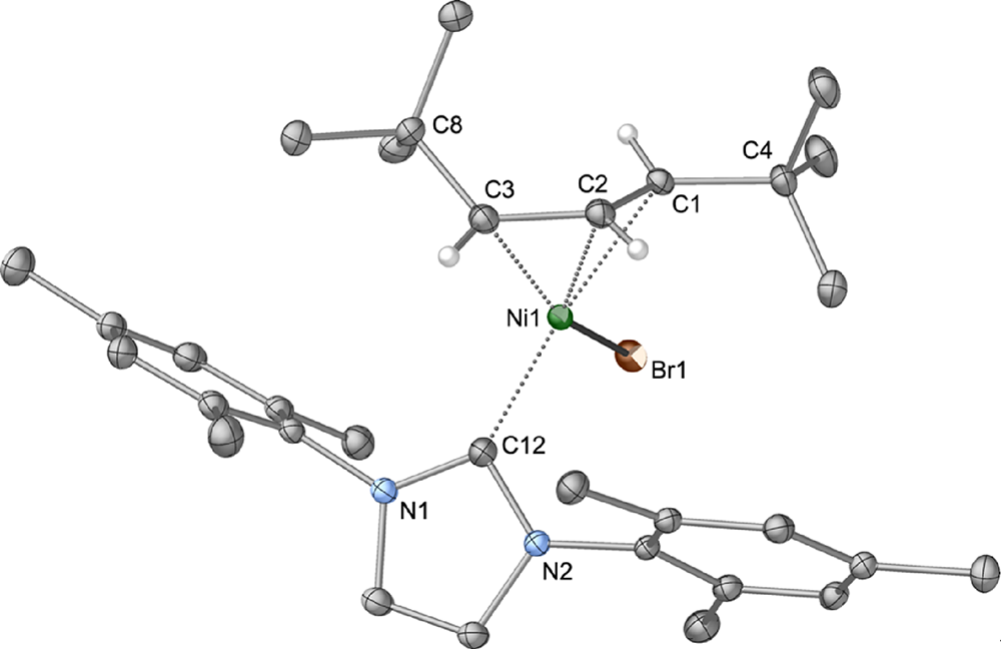
Thermal ellipsoid plot (50% level) of the structure of **5**. For clarity, all hydrogen atoms except those on the C_3_ fragment of the allyl ligands are removed, and the others are given arbitrary radii. Selected bond distances (Å) and angles (deg): Ni1–Br(1), 2.3461(3); Ni(1)–C(1), 2.1132(15); Ni(1)–C(2), 1.9671(15); Ni(1)–C(3), 2.0463(15); Ni(1)–C(12), 1.9409(15); C(1)–C(2), 1.399(2); C(2)–C(3), 1.419(2); C(1)–C(4), 1.524(2); C(3)–C(8), 1.545(2); C(12)–Ni(1)–Br(1), 95.37(4); C(1)–C(2)–C(3), 121.46(14); C(4)–C(1)–C(2)–C(3), 177.18(14); C(1)–C(2)–C(3)–C(8), −44.4(2).

Complex **5** crystallizes from hexanes as a monomer, with an η^3^-coordinated [A^2t^] ligand and terminally bonded bromide and IMes ligands. The *t*-Bu groups on the [A^2t^] ligand are in a *syn*, *anti-*relationship (Figure 11), as is found in the solution. The Ni–C bond lengths range from 1.9671(15) to 2.1132(15) Å, which are comparable to those in other [(allyl)NiX(NHC)] complexes.^57,73−81^ In [(C_3_H_5_)NiBr(Me_4_Im)] (Me_4_Im = 1,3,4,5-tetramethylimidazol-2-ylidene), for example, the corresponding distances are 1.984(4) to 2.056(3) Å.^75^ Similarly, the Ni–Br and Ni–NHC distances of 2.3461(3) and 1.9409(15) Å in **5** can be compared to the analogous lengths of 2.3856(7) and 1.907(2) Å, respectively, in [(C_3_H_5_)NiBr(Me_4_Im)]. As is typical for similar configurations with TMS substituents,^13^ the central carbon atom in the *syn t*-Bu group (C4) is close to the C_3_ plane (0.09 Å), whereas the center of the *anti t*-Bu group (C8) is considerably displaced from the allyl plane (0.89 Å).

As a point of comparison, the trimethylsilyl analogue of **5**, i.e., [A′Ni(IMes)Br] (**6**), was synthesized and characterized. The same synthetic protocol was used (i.e., [A′NiBr] + IMes in toluene), generating a brick-red solid with identical solubility and similar solid-state stability. Its NMR spectrum is also consistent with a *syn*, *anti*-configuration of the TMS substituents on the allyl ligand, displaying the same fast/slow rotations. No minor products are formed in this reaction. This is not unreasonable, as a substantial amount of [{A′NiBr}_2_] is expected to be present in its *syn, anti*-conformation at equilibrium, making it much less likely for a *syn, syn* adduct to form and become trapped.

**Figure 12:**
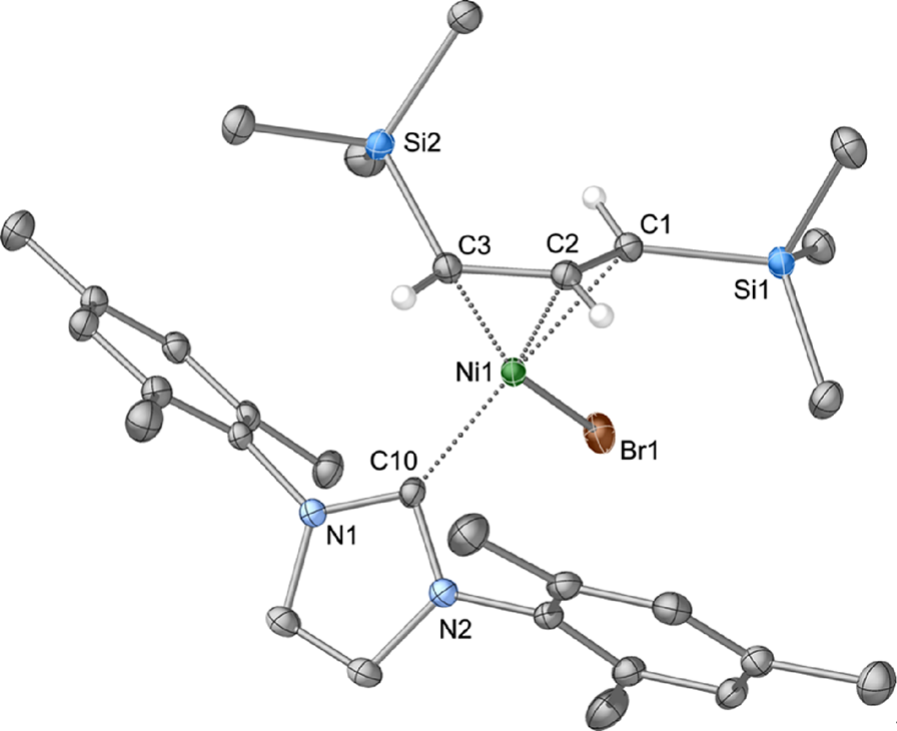
Thermal ellipsoid plot (50% level) of the structure of **6**. For clarity, all hydrogen atoms except those on the C_3_ fragment of the allyl ligands are removed, and the others are given arbitrary radii. Selected bond distances (Å) and angles (deg): Ni1–Br(1), 2.3333(3); Ni(1)–C(1), 2.095(2); Ni(1)–C(2), 1.976(2); Ni(1)–C(3), 2.025(2); Ni(1)–C(10), 1.923(2); C(1)–C(2), 1.404(2); C(2)–C(3), 1.425(2); C(1)–Si(1), 1.868(2); C(3)–Si(2), 1.888(2); C(12)–Ni(1)–Br(1), 93.99(5); C(1)–C(2)–C(3), 119.00(15); C(3)–C2)–C(1)–Si(1), −175.17(12); C(1)–C(2)–C(3)–Si(2), −44.3(2).

The complex crystallizes from hexanes as a monomer, with an η^3^-coordinated [A′] ligand and terminally bonded bromide and IMes ligands. The TMS groups on the A^′^ ligand are in a *syn*, *anti-*relationship. As the compound is isostructural with the A^2t^ analogue **5**, it will be discussed only briefly (Figure 12). All the bond lengths to Ni are within ∼0.02 Å of those found in **5** but are generally shorter. Even the displacements of the TMS or *t*-Bu groups on the allyl ligands from the respective C_3_ planes are almost the same, as reflected in the closely similar torsion angles, differing at most by 2°. The steric bulk of the TMS group relative to *t*-Bu has not introduced any significant distortions into their respective complexes, consistent with their being effectively isosteric substituents (see below).

A previous study of the allyl coupling product {A′}_2_ generated from solution reactions found that it was generated in two diastereomeric forms of *C*_i_ (*meso*) and *C*_2_ (*rac*) symmetry.^13^ A single crystal X-ray structure of the compound was severely disordered, rendering the exact conformation of the molecule uncertain. A higher quality structure of the *meso* form was obtained recently that displays approximate inversion symmetry.^82^

**Figure 13:**
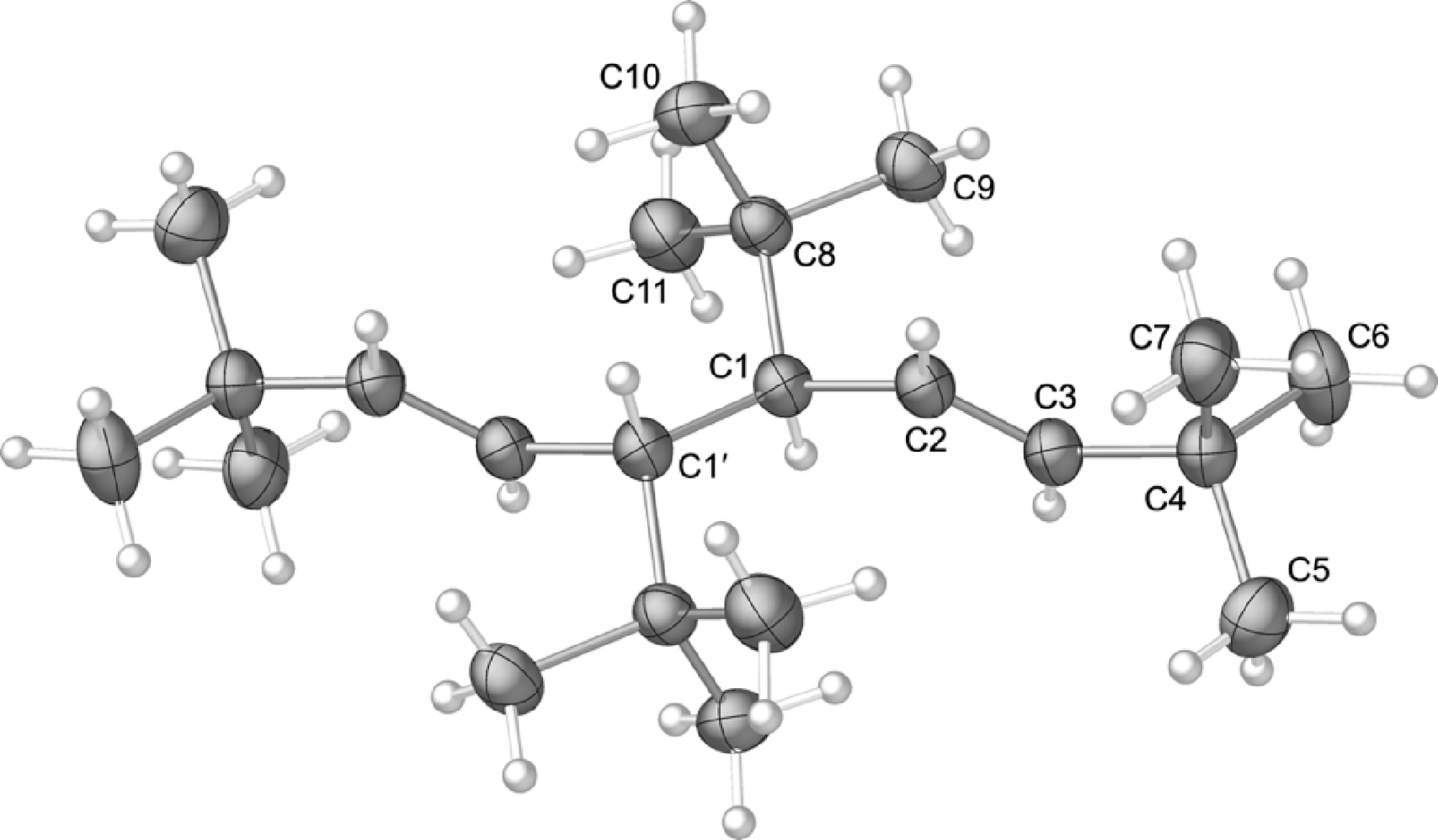
Thermal ellipsoid plot (30% level) of the structure of *meso*-{A^2t^}_2_ (**7**); hydrogen atoms are given arbitrary radii. Selected bond distances (Å) and angles (deg): C1–C1′, 1.571(3); C1–C2, 1.506(2); C2–C3, 1.326(3); C3–C4, 1.507(2); C1–C8, 1.586(2); C1′–C1–C2, 111.37(16); C1–C2–C3, 125.21(16); C2–C3– C4, 128.52(17).

The related compound {A^2t^}_2_ (**7**) was produced during the present experiments, including some in highly crystalline form from hexanes. Compound **7** crystallizes in the triclinic space group *P*1̅, and comprises a single diastereomer, the *meso* form (Figure 13). The molecule lies on a crystallographic inversion center, and thus only one-half of the molecule is unique. The structure is largely as would be expected; the central C1–C1′ bond, at 1.571(3) Å, exactly matches the comparable distance found in [(^t^BuMe_2_Si)CH(CH = CHSiMe_2_^t^Bu)]_2_ (1.571(4) Å), for example, which was ascribed to steric crowding between the bulky alkylsilyl groups.^83^ Even with their shorter C–C bonds compared to the C–Si bonds in the *t*-butyldimethylsilyl species, the *t*-Bu groups in **7** evidently exert similar steric pressure.

DFT calculations at the B3PW91-D3BJ/def2-TZVP level indicate that the observed *meso* form of **7** (*C*_i_) is 22 kJ kcal mol^–1^ lower in energy than a hypothetical *rac* (*C*_2_) configuration.

## Discussion

The scarcity of complexes containing *t*-Bu substituted allyl ligands is related, at least in part, to the lack of efficient synthetic routes. Even so, it is worth comparing the properties of such ligands with the closely related TMS-substituted versions, which are currently more accessible.

**Figure 14:**
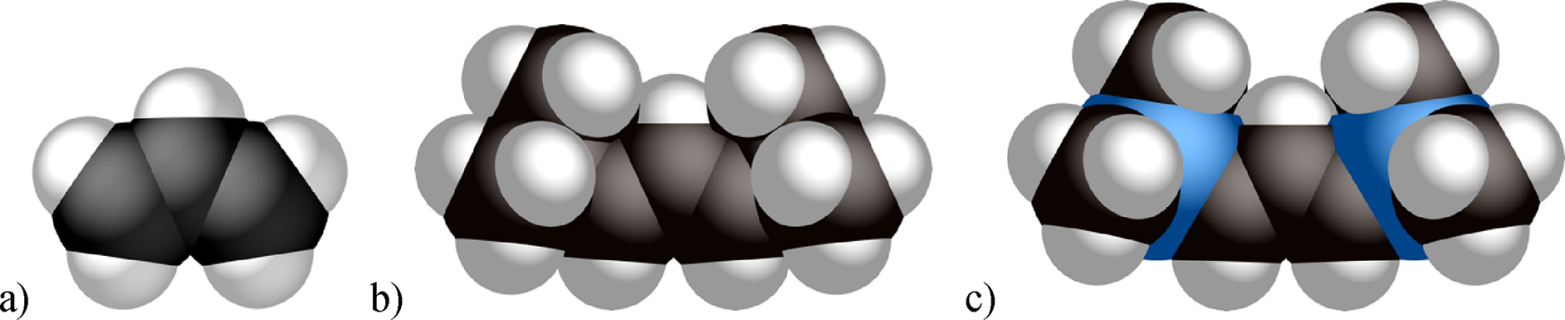
Relative van der Waals volumes of three allyl anions: (a) parent [C_3_H_5_] (57 Å^3^); (b) [A^2t^] (195 Å^3^); (c) [A′] (231 Å^3^); the latter two were calculated with *syn*, *syn* substituents.

Given the larger radius of silicon relative to carbon, it is not surprising that the calculated van der Waals volume of the [A′] anion is larger than [A^2t^]. Relative to the unsubstituted [C_3_H_5_] anion (57 Å^3^), the [A^2t^] anion is approximately 3.4 times larger (195 Å^3^), whereas [A′] (231 Å^3^) is 4.1 times larger; equivalently, [A′] is about 18% larger than [A^2t^] (Figure 14).^84^

**Figure 15:**
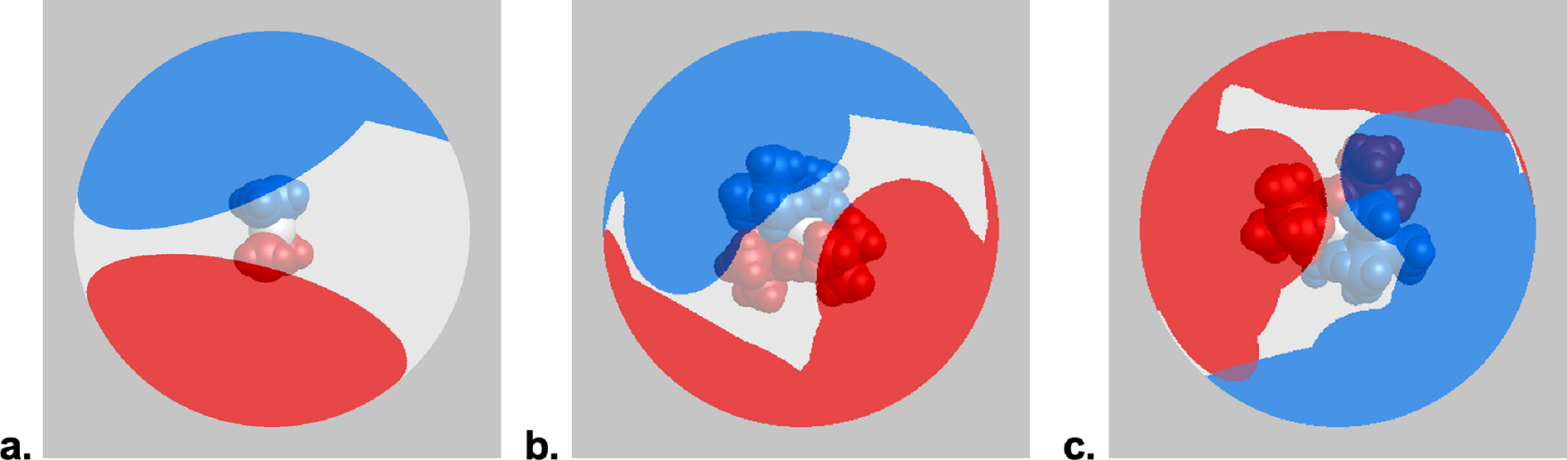
Visualization of the extent of coordination sphere coverage (G_complex_) of: (a) [Ni(C_3_H_5_)_2_], 70.4%; (b) [NiA^2t^_2_], 90.8%; (c) [NiA′_2_], 92.9%. Optimized coordinates (B3PW91-D3BJ/def2-TZVP on all atoms, *C*_2_ symmetry) were used with the program Solid-G.^85^ The G_complex_ value represents the net coverage, so that regions of the coordination sphere where the projections of the ligands overlap are counted only once.

However, the difference in vdW volumes does not translate into proportional changes in the metal coordination sphere shielding. As estimated with the program Solid-G,^85^ and specifically by the value of *G*(*L*), the percentage of the metal coordination sphere shielded by particular ligands, the numbers for [A^2t^] and [A′] ligands are quite similar. For example, in the isostructural [{(allyl′)NiBr}_2_] dimers, *G*(*L*) = 41.6% and 42.2% for the [A^2t^] and [A′] ligands, respectively. The comparable *G*(*L*) values for the mono(allyl) complexes **5** and **6** ([(IMes)NiBr(allyl′)]) are 44.0% and 44.2%, respectively. Crystallographically derived coordinates were used for input in these calculations, which are subject to some artifacts (e.g., the well-known shortening of C–H bond lengths).^86,87^ To remove such effects and the influence of other ligands on the (allyl′)–Ni bonding, DFT calculations were used to optimize the structures of [Ni(C_3_H_5_)_2_],^88^ [NiA′_2_],^13^ and (the as yet unknown) [NiA^2t^_2_], with the latter two calculated with *syn*, *anti*-substituents. For the three complexes, the solid-*G*′s G_complex_ values, which are the net coverage of the metal coordination sphere by all the ligands, are 70.4, 90.8, and 92.9%, respectively (Figure 15).

These results suggest that the two ligands [A′] and [A^2t^] are nearly isosteric, a conclusion that comports with the similar structural parameters found in related compounds, e.g., **4** and [{A′NiBr}_2_], and the IMes derivatives **5** and **6**. Differences in their coordination chemistry therefore must originate primarily from electronic effects, i.e., in the distinctions between purely hydrocarbyl and organosilyl-based compounds. For example, calculations at the B3PW91-D3BJ/def2TZVPD level of Δ*H*° for the reaction HA → [A] + H^+^, where HA is the relevant propene, demonstrate that HA′ is more easily deprotonated than HA^2t^: Δ*H*° = +389 kcal mol^–1^ and +367 kcal mol^–1^ for HA^2t^ and HA′, respectively.^89^

These electronic effects were also examined in the context of molecular species. This was done by comparing analogous complexes of the parent allyl ligand [C_3_H_5_], [A^2t^], and [A′]. CO stretching frequencies were calculated for the geometry-optimized complexes of the form [A^x^Ni(CO)_2_Br]. [(C_3_H_5_)Ni(CO)_2_Br] has been studied as an intermediate in the carbonylation reaction of allyl bromide catalyzed by Ni(CO)_4_,^90^ and [(2-methylallyl)Ni(CO)_2_Br] has been reported via the reaction of [{(2-methylallyl)NiBr}_2_] with carbon monoxide, although the species could not be isolated.^91,92^ The calculated stretching frequencies are included below in Table 3. Even though the trimethylsilyl group is sometimes considered electronically analogous to a proton,^93^ [A′] was found to be substantially more electron donating than the parent allyl or 2-methylallyl. As expected, the [A^2t^] complex was found to be the most electron-donating of the ligands studied, but the difference in the IR frequencies between it and [A′] was approximately half the difference between the C_3_H_5_ parent and [A′] derivative. This suggests that their behaviors would be expected to be generally similar, except in particularly sensitive systems.

**Table 3:** Calculated CO Stretching Frequencies for a Set of [A^x^Ni(CO)_2_Br] Compounds

**complex**	**calculated CO frequencies (cm**^**–1**^**)**^a^
[(C_3_H_5_)Ni(CO)_2_Br]	2088, 2058
[(2-methylallyl)Ni(CO)_2_Br]	2085, 2055 (exp. 2083, 2049)^91^
[A′Ni(CO)_2_Br]^b^	2068, 2037
[A^2t^Ni(CO)_2_Br]^b^	2059, 2030

a Geometries were optimized and frequencies calculated at the PW91PW91/def2TZVP level.

b The A′- and A^2t^-ligated complexes were optimized with *syn,syn* geometries.

Although [{A^2t^NiBr}_2_] fits into the well-explored class of [{(allyl)NiX}_2_] complexes, some details of its synthesis and reactivity were unexpected and deserve additional comment. For example, the reaction of A^2t^Br with Ni(0) complexes is atypical, starting with the way that A^2t^Br induces decomposition of Ni(COD)_2_. In the recovered organic material from these reactions, only A^2t^Br and COD are present in meaningful amounts, eliminating most sequential oxidation/reduction pathways from consideration. A^2t^Br likely acts as an olefinic ligand, binding the nickel center in competition with COD, perhaps forming an unstable “[(COD)NiBr(A^2t^)]” complex that subsequently decomposes to the olefins and nickel metal. However, the decomposition rate does not noticeably decrease upon adding excess COD, which might be expected to stabilize such a mixed ligand species. The detailed mechanism of the decomposition, therefore, remains indeterminate.

Even once isolated, the extent to which [{A^2t^NiBr}_2_] resists adduct formation is atypical. The formation of ligand adducts from [{(allyl)NiX}_2_] complexes is well-precedented, and the [(allyl)NiLX] archetype has generally been found to be more stable than the bridged bimetallic species.^57,77,94^ In the case of [{A^2t^NiBr}_2_], however, stable binding is only observed with the highly basic IMes ligand, and even this is accompanied by a decrease in solution-phase stability. The reactivity of [{A^2t^NiBr}_2_] with triphenylphosphine was distinct even from the very similar [{A′NiBr}_2_], and so this reaction was studied computationally.

**Figure 16:**
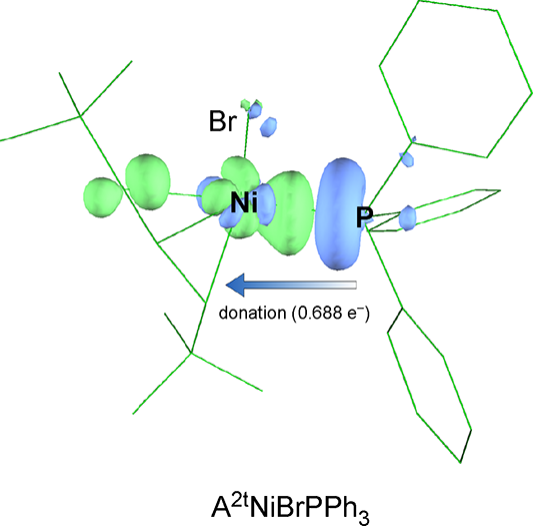
Blue and green isosurfaces display the region where the electron density decreases and increases, respectively, owing to the orbital interaction described by NOCV pair 1 (Δ*E*_1_^orb^) in [A^2t^NiBrPPh_3_]. The closely similar figure for [A′NiBrPPh_3_] is found in the SI (Figure S20).

ETS-NOCV (extended transition state–natural orbitals for chemical valence) fragmentation analysis on both [A^2t^Ni(PPh_3_)Br] and [A′Ni(PPh_3_)Br] was conducted to evaluate the relative strength of the Ni–P interaction in each complex (Figure 16).^95^ The sum of all NOCV pairs (Δ*E*^orb^) is −63.7 and −64.7 kcal mol^–1^ for [A^2t^Ni(PPh_3_)Br] and [A′Ni(PPh_3_)Br], respectively, indicating that the orbital interaction stabilizes both complexes nearly equally. The Ph_3_P→NiBr(allyl) donation is by far the most energetically significant interaction, equaling 39.0 kcal mol^–1^ for [A′Ni(PPh_3_)Br] and 37.6 kcal mol^–1^ for [A^2t^Ni(PPh_3_)Br]. For comparison, the next most important interaction for either molecule is <8 kcal mol^–1^. The binding affinity of nickel for triphenylphosphine is essentially as high for the [A^2t^NiBr] monomer as the [A′NiBr] monomer. This slight difference is also reflected in the Mayer bond orders of 0.84 and 0.80 for the Ni–P bonds in [A′Ni(PPh_3_)Br] and [A^2t^Ni(PPh_3_)Br], respectively. This suggests that the difference might lie earlier in the adduct formation, i.e., in the rearrangement of the *syn*, *syn*-conformations found in the [{A^x^NiBr}_2_] dimers into the *syn, anti*-arrangements in the PPh_3_ adducts. Although the activation energies for these changes are currently unknown, the energies of *syn, anti*-dimers relative to the *syn*, *syn*-versions are higher for the [A^2t^] complex than the [A′] counterpart (see the SI). Even though all the steric and electronic differences between the [A^2t^Ni(PPh_3_)Br] and [A′Ni(PPh_3_)Br] complexes are relatively small, they all point in the same direction, and their cumulative effect may well be to inhibit PPh_3_ binding in the [A^2t^Ni(PPh_3_)Br] complex. The greater basicity of IMes (and other phosphines^96^) could reasonably overcome these differences, and hence, ligand dissociation is not observed in their solutions.

## Conclusions

Although the volumes of the [1,3-(*t*-Bu)_2_C_3_H_3_] (= [A^2t^]) and [1,3-(TMS)_2_C_3_H_3_] (= [A′]) anions are different, in metal complexes the ligands appear to be functionally isosteric. The dissimilarities in their coordination chemistry therefore must be primarily electronic in origin. With no readily available alkali metal salt for the [A^2t^] ligand, synthesis of its metal complexes currently relies on customized approaches. In the case of nickel, [{A^2t^NiBr}_2_] proves to be a versatile starting material for substitution reactions. It shares similarities with its trimethylsilyl counterpart, particularly in the structures of its complexes, but their behavior is not exactly parallel. For example, [{A^2t^NiBr}_2_] was found to be atypically resistant to interacting with PPh_3_, forming only a weakly bound complex. This reluctance could, however, be useful for applications in which ligand dissociation is desirable, such as catalysis of addition-type olefin polymerization (e.g., a nickel complex with weakly binding SbPh_3_ ligands was found to be uniquely effective in polymerizing silylnorbornenes).^107^ We are continuing to examine the distinctive properties of this understudied allyl variant.

## Experimental Section

### General Considerations

Unless noted otherwise, all syntheses were conducted under rigorous exclusion of air and moisture using Schlenk line and glovebox techniques. Proton (^1^H), carbon (^13^C), and phosphorus (^31^P) NMR spectra were obtained at ambient temperature on a Bruker AV-400 MHz spectrometer at 400, 100, and 162 MHz, respectively. Proton and carbon spectra were referenced to the residual resonances of C_6_D_6_, toluene-*d*_8_, DMSO-*d*_6_, and CDCl_3_; phosphorus (^31^P) spectra were referenced to external H_3_PO_4_. Structural assignments were made using additional information from gCOSY and gHSQC experiments.

### Materials

Pivaldehyde, pinacolone, neopentyl iodide, triphenylphosphine, lithium hexamethyldisilazane (LiHMDS) in THF, and sodium borohydride were purchased from Oakwood Products. Thionyl bromide, thionyl chloride, and potassium metal were purchased from Sigma-Aldrich. Bis(cyclooctadiene)nickel(0) was purchased from Strem and used as received. The literature method was used to prepare A^2t^Br^42^ and A^2t^Cl.^26^ Anhydrous inhibitor-free tetrahydrofuran (THF), toluene, and diethyl ether were dried by passage through activated alumina and then stored over 4A molecular sieves before use. Before use, hexanes were distilled under nitrogen in the presence of sodium metal/benzophenone. Deuterated benzene and chloroform were purchased from Cambridge Isotopes and stored over 4A molecular sieves. Deuterated toluene was purchased from Sigma, degassed before use, and stored over 4A molecular sieves.

### Mechanochemical Protocol

Planetary milling was performed with a Retsch PM100 mill, 50 mL stainless steel grinding jar type C, and a safety clamp for air-sensitive grinding. Mixer milling was performed with a Retsch model MM200 mill. Ball milling reactions used Formtech Smartsnap stainless steel milling jars with two stainless steel (440 grade) ball bearings (^5^/_16_ in (8 mm), 2.0 g each) that were thoroughly cleaned with detergent and water, then washed with acetone, and dried in a 125 °C oven before use.

### Preparation of 2,2,6,6-tetramethylhept-4-en-3-one (1)

#### Method A

An oven-dried 500 mL Schlenk flask was equipped with a Teflon stirring bar and placed in a nitrogen-filled glovebox. THF (100 mL) was added to the flask, and the opening was sealed with a ribbed septum. The flask was removed from the glovebox and placed on a Schlenk line. Pinacolone (7.43 mL, 59.4 mmol, 1 equiv) was added to the flask via syringe. The THF solution was chilled to −78 °C for 10 min before manipulation. An oven-dried 125 mL flask was filled with 60 mL of 1.0 M LiHMDS in THF and sealed with a ribbed septum. The LiHMDS was transferred using a cannula into the reaction flask, and the solution was stirred for 15 min at −78 °C. Pivaldehyde (7.10 mL, 65.4 mmol, 1.1 equiv) was added to the reaction via syringe. The ice bath was removed from the reaction flask and the mixture stirred at room temperature overnight. After the reaction was complete, 50 mL of saturated ammonium chloride and 50 mL of diethyl ether were added. The mixture was transferred to a 500 mL separatory funnel, and the layers were separated. The organic layer was washed twice with 50 mL portions of deionized water. The aqueous washes were collected and extracted with 50 mL of diethyl ether. The organics were then combined, washed with 100 mL of brine, separated, and dried over magnesium sulfate before being filtered into a 500 mL round-bottom flask.

The volatiles were removed via rotary evaporator to leave a yellow/amber liquid. The liquid was transferred to an Erlenmeyer flask. Cold methanol (−78 °C) was added to the flask slowly to promote recrystallization. The methanol/crude product mixture was placed in the ice bath, and methanol was added until a roughly 1:1 mixture was obtained. The product was obtained as a white powder and recovered by filtration. The product was then placed in vials and placed under a high vacuum to remove any residual methanol and other volatile impurities. The product was identified by its characteristic ^1^H NMR spectrum.^26^ Yield: 6.9 g (69%).

#### Method B

Pinacolone (1.49 mL, 11.9 mmol, 1.0 equiv), pivaldehyde (1.29 mL, 11.9 mmol, 1.0 equiv), and sodium hydroxide (476 mg, 11.9 mmol, 1.0 equiv) were evenly distributed into two 15 mL Formtech Smartsnap stainless steel milling jars with two 8 mm stainless steel ball bearings per jar. The jars were closed, and the reaction was milled for 30 min at 30 Hz. The jars were then opened, revealing a slurry. The reaction mixture was treated with 8 mL of 10% HCl (2 mL per jar section) and combined in a 125 mL Erlenmeyer flask. The jars were rinsed with 20 mL of diethyl ether (5 mL per jar section). Organic and aqueous portions were combined in a 125 mL separatory funnel. The aqueous layer was removed and extracted with an additional 10 mL of diethyl ether. The organics were combined, washed with 5 mL of brine, separated, and dried over magnesium sulfate before filtration into a 100 mL round-bottom flask. The volatiles were removed by rotary evaporation, and the crude product was placed on a high vacuum to remove any remaining volatile impurities. The product was isolated with no further purification. The product was identified by its characteristic NMR spectrum.^26^ Yield: 1.04 g (52%).

Both methods often generate double-addition products as minor products in varying amounts. For example, 5-(*t*-Bu)-2,2,8,8-tetramethylnonane-3,7-dione is inevitably found under conditions of excess pinacolone, and occasionally observed even when the ketone is the limiting reagent. When pivaldehyde is used in excess, 5-hydroxy-2,2,6,6-tetramethylheptan-3-yl pivalate (**3**) is frequently formed, forming X-ray quality crystals. Only one set of peaks is observed in the proton NMR spectrum; taken in conjunction with the crystal structure, this suggests that only the *anti*-enantiomers are formed. For *anti*-5-hydroxy-2,2,6,6-tetramethylheptan-3-yl pivalate: ^1^H NMR (400 MHz, CDCl_3_, 298 K): δ 0.86 (s, 9H, C(CH_3_)_3_); 0.91 (s, 9H, C(CH_3_)_3_); 1.21 (s, 9H, C(CH_3_)_3_); 1.52 (m, 2H, methylene CH_2_); 2.81 (irreg d, 4.30 Hz, 1H, OH); 2.88 (m, 1H, OC(*H*)*t*-Bu); 4.84 (m, 1H, C(*H*)OH) (Figure S1).

### 2,2,6,6-Tetramethylhept-4-en-3-ol

A 250 mL round-bottom flask was equipped with a stirring bar. The flask was charged with 2,2,6,6-tetramethylhept-4-en-3-one (8.00 g, 47.5 mmol, 1 equiv) and methanol (50 mL). The solution was cooled to 0 °C in an ice bath, and sodium borohydride (1.80 g, 47.5 mmol, 1 equiv) was added slowly to the flask. The reaction was stirred at 0 °C for 60 min after the last addition of sodium borohydride. The reaction was kept cool, and 10 mL of saturated Rochelle’s salt solution was slowly added to quench the reaction mixture. The quenched solution was diluted with 40 mL of diethyl ether and filtered into a 250 mL round-bottom flask. The volatiles were removed to give a pale-yellow emulsion. The material was dried over sodium sulfate to remove all water from the product. The product was transferred with a pipet into a vial and stored without further purification. The product was identified by its characteristic ^1^H NMR spectrum.^96^ Yield: 5.05 g (63%).

### 3-Bromo-2,2,6,6-tetramethyl-4-heptene

The compound was prepared as described in the literature.^42^ The proton NMR spectrum of this molecule in aromatic solvents differs somewhat from its spectrum in CDCl_3_, so these shifts are included here. ^1^H NMR (400 MHz, C_6_D_6_, 298 K): δ 0.87 (s, 9H, C(CH_3_)_3_); 0.96 (s, 9H, C(CH_3_)_3_); 4.24 (d, 10.36 Hz, 1 H, sp^3^ C–H); 5.38 (d, *J* = 15.5 Hz, 1 H, sp^2^ C(*t*-Bu)-H); 5.68 (dd, *J* = 15.5, 10.4 Hz, 1 H, center sp^2^ C–H). ^13^C{^1^H} NMR (151 MHz, C_6_D_6_, 298 K): δ 144.0 (s, center vinyl carbon); 124.4 (s, terminal vinyl C(H)*t*-Bu); δ 69.9 (s, BrC(H)*t*-Bu); 35.5 (s, *t*-Bu CMe_3_); 32.4 (s, *t*-Bu CMe_3_); 29.0 (s, *t*-Bu (CH_3_)_3_); 27.1 (s, *t*-Bu (CH_3_)_3_).

### 5-(*t*-Butyl)-2,2,8,8-tetramethylnonane-3,7-dione (**2**)

A 1 L flask with a 24/40 addition arm attachment was flame-dried, joints greased, and assembled, and a Teflon stirring bar was added before being flushed with nitrogen gas on a Schlenk line. A separate oven-dried 500 mL Schlenk flask was placed in a nitrogen-filled glovebox and filled with 200 mL of dry diethyl ether. The flask was removed from the glovebox and placed on a Schlenk line. The diethyl ether was transferred via cannulation. Pinacolone (22.3 mL, 178 mmol, 1.8 equiv) was added to the THF and stirred. The resulting solution was cooled to −78 °C and LiHMDS (1.0M, 178 mL, 1.8 equiv) was added to the flask. The solution was stirred at low temperature for 15 min. Pivaldehyde (10.6 mL, 97.6 mmol, 1 equiv) was added to the flask, and the reaction was removed from the ice bath and stirred at room temperature overnight. The reaction was quenched with 200 mL of saturated ammonium chloride and the flask contents were transferred to a 1 L separatory funnel. The organics were washed twice with two 100 mL portions of deionized water, and the washes were combined. The aqueous washes were then extracted twice with 60 mL portions of diethyl ether. The organics were combined, washed with brine, and dried over magnesium sulfate. The dried organics were filtered into a 1 L flask and concentrated with a rotary evaporator to give a viscous amber oil. The oil slowly crystallized when agitated with a pipet to give light tan needles. The product was obtained without further purification (11.7 g, 45%), and was identified by its characteristic ^1^H NMR spectrum.^44^^1^H NMR (400 MHz, CDCl_3_, 298 K): δ 0.86 (s, 9H, C(CH_3_)_3_); 1.14 (s, 18H, C(CH_3_)_3_); 2.26 (dd, 2H, *J* = 17.01 Hz, 7.91 Hz, C–H_methylene_); 2.49 (m, 1H, C–H_methyne_); 2.67 (dd, 2H, *J* = 16.95 Hz, 3.93 Hz, *J* = 1.62, C–H_methylene_). ^13^C{^1^H} NMR (100 MHz, C_6_D_6_, 298 K): δ 27.08 (CH_3_), 27.70 (CH_2_); 33.56 (CMe_3_); 37.67 (CMe_3_); 38.33 (CMe_3_); 44.61 (CH(*t*-Bu)); 215.54 (C_sp2_-O) (Figures S2 and S3).

### Synthesis of Allylnickel Complexes

#### [{A^2t^NiBr}_2_] (**4**)

The synthesis was performed in a nitrogen-filled glovebox. A^2t^Br (0.179 g, 0.767 mmol) and acetonitrile (0.303 g, 7.38 mmol) were combined in a glass vial, then dissolved in 3–5 mL THF. In a round-bottom flask equipped with a magnetic stir bar, Ni(COD)_2_ (0.212 g, 0.771 mmol) was dissolved in 3–5 mL THF. The A^2t^Br solution was then added dropwise to the Ni(COD)_2_ solution, during which the mixture gradually darkened to a deep red color. This solution was allowed to stir for 30 min, during which time a black solid was deposited at the bottom of the flask. The solution was then filtered through a fine glass frit, and the volatiles were removed by vacuum. The resulting red solid was then dissolved in 5 mL acetonitrile, followed by extraction with 3 × 3 mL portions of hexanes. The hexane washes were discarded, and the acetonitrile solution evaporated under vacuum, leaving spectroscopically pure [{A^2t^NiBr}_2_] (0.173 g, 77%; yields in repeated reactions consistently ranged from 65–80%) as a chalky red, light powder. The solid is sparingly soluble in nonpolar solvents and can be stored indefinitely in the glovebox. ^1^H NMR (400 MHz, DMSO-*d*_6_): δ 5.16 (t, *J* = 12.0 Hz, 1H, center allyl proton); 2.82 (d, *J* = 12.0 Hz, 2H, allyl *anti* C(H)*t*-Bu); 1.02 (s, 18 H, *t*-Bu (CH_3_)_3_). ^1^H NMR (400 MHz, C_6_D_6_): δ 4.94 (t, *J* = 12.3 Hz, 1H, center allyl proton); 2.46 (d, *J* = 12.3 Hz, 2H, allyl *anti* C(H)*t*-Bu); 1.11 (s, 18 H, *t*-Bu (CH_3_)_3_). ^13^C{^1^H} NMR (151 MHz, C_6_D_6_): δ 96.8 (s, center allyl carbon); 84.2 (s, allyl terminal C(H)*t*-Bu); 33.4 (s, *t*-Bu Cme_3_) 29.4 (s, *t*-Bu (CH_3_)_3_). Despite repeated attempts, satisfactory elemental analysis could not be obtained for this compound (Figures S4, S5).

#### Improved synthesis of [{A′NiBr}_2_] from [NiA′_2_]

In a nitrogen-filled glovebox, a 10 mL Schlenk flask was charged with 10.0 mL dry hexane. This flask was placed on a Schlenk line, and Br_2_ (0.21 mL, 4.08 mmol) was added, and the flask was swirled until well-mixed. A separate 50 mL Schlenk flask was charged with a magnetic stir bar and [NiA′_2_,] (0.637 g, 1.48 mmol) which was then dissolved in 25–30 mL hexane. This flask was placed on the Schlenk line and cooled to −78 °C. A plastic syringe was used to add the Br_2_/hexane solution (3.60 mL, 1.44 mmol) dropwise to the [NiA′_2_] solution. The reaction mixture slowly changed from dark orange to deep scarlet. The solution was then allowed to warm to room temperature overnight. The flask was brought into the glovebox, the mixture was filtered through a glass frit, and the filtrate solution was concentrated under a vacuum. The concentrated hexane solution was extracted three times with acetonitrile, losing most of its color in the process. The combined acetonitrile extracts were then extracted twice with hexane, and the volatiles removed under vacuum. Spectroscopically pure [{A′NiBr}_2_] was isolated in 62% yield (0.297 g). Higher yields have been reported using the previously published methods,^13^ but unrecognized impurities likely inflate these numbers, and the present method is substantially more consistent.

Two previously unreported species are present in the product mixture, believed to be the eclipsed and staggered isomers of *syn*,*anti–*[{A′NiBr}_2_]. New product **1** is always in excess compared to new product **2**, generally by a ratio of 2–4:1. One or both of these species have been identified as minor products in all known previously recorded spectra of [{A′NiBr}_2_], in ratios from 0.5 to 1 (combined new products):1 *syn*,*syn*-[{A′NiBr}_2_]. However, the new procedure outlined here initially produces these products in much larger amounts, as great as 2:1 compared to *syn*, *syn*-[{A′NiBr}_2_]. It is thought that transient binding of trace acetonitrile leftover from the new extraction process promotes a *syn*,*anti*-configuration, in line with all known [A′Ni(L)Br] complexes, which display *syn*,*anti*-configurations. Interestingly, the relative amount of the new products decreases over time, eventually reaching as low as 0.30:1; this might result from evaporation of the leftover acetonitrile, followed by slow isomerization to the thermodynamically favored *syn*, *syn* form.

New product **1**^1^H NMR (400 MHz, C_6_D_6_, 298 K): δ 5.58 (d, *J* = 14.8, 9.2 Hz, 1H, center allyl proton); 2.99 (d, *J* = 9.2 Hz, 1H, allyl *syn*-C(H)TMS); 2.06 (d, *J* = 14.78, 1 H, allyl *anti* C(H)TMS). New product **2**^1^H NMR (C_6_D_6_): δ 5.56 (dd, *J* = 15.3, 9.4 Hz, 1H, center allyl proton); 2.91 (d, *J* = 9.4 Hz, 1H, allyl *syn*-C(H) TMS); 1.99 (d, *J* = 15.3 Hz, 1 H, allyl *anti*-C(H)TMS). Unfortunately, the TMS CH_3_ protons appear to overlap with each other or with trace impurities and, therefore, cannot be confidently assigned (Figure S6).

#### Synthesis of [A^2t^Ni(PPh_3_)Br]

In a nitrogen-filled glovebox, [{A^2t^NiBr}_2_] (0.016 g, 0.027 mmol) was placed in a glass vial and dissolved in 5 mL THF. Solid PPh_3_ (0.014 g, 0.053 mmol) was added to the solution. The PPh_3_ quickly dissolved with no change in the color of the solution. The solution was then filtered through a Celite-packed pipet filter, and the volatiles were removed by vacuum to yield a hard, dark red paste. NMR analysis shows this material, once dissolved, comprises a mixture of [A^2t^Ni(PPh_3_)Br], [{A^2t^NiBr}_2_], and free triphenylphosphine (see Table S1 for ratio details). Anal. calcd. for C_29_H_36_BrNiP: C, 62.85; H, 6.55. Found: C, 60.90; H, 6.23. The low value for carbon likely reflects the solid-state decomposition of the adduct. ^1^H NMR (600 MHz, d_8_-toluene, 298 K): δ 7.78 (br s, 6H, PPh_3_ Ar–H); 7.03 (m, 9H, PPh_3_ Ar–H); 4.86 (dd, *J* = 14.6, 8.7 Hz, 1 H, center allyl CH); 4.18 (br m, 1 H, allyl *anti* C(H) *t*-Bu); 3.14 (br m, 1 H, allyl *syn* C(H) *t*-Bu); 1.48 (s, 9 H, *t*-Bu (CH_3_)_3_; 0.93 (s, 9 H, *t*-Bu (CH_3_)_3_). ^1^H NMR (600 MHz, d_8_-toluene, 253 K): δ 7.78 (br m, 6H, PPh_3_ Ar–H); 7.00 (br m, PPh_3_ Ar–H); 4.82 (dd, *J* = 14.34, 8.75 Hz, 1 H, center allyl CH); 4.20 (dd, *J* = 14.3, 8.9 Hz, 1 H, allyl *anti* C(**H**)*t*-Bu); 3.12 (br m, 1 H, allyl *syn* C(H) *t*-Bu); 1.52 (s, 9 H, *t*-Bu (CH_3_)_3_; 0.92 (s, 9 H, *t*-Bu (CH_3_)_3_). ^13^C{^1^H} NMR (151 MHz, toluene-*d*_8_, 298 K): δ 134.5 (bd, *J* = 8.4 Hz, PPh_3_ C–H); 133.1 (bd, *J* = 34.8 Hz, PPh_3_ ipso C); 129.7 (br s, PPh_3_ para C–H); 128.1 (vb s, PPh_3_ C–H); 98.3 (s, allyl center C–H); 96.9 (bd, *J* = 20.0 Hz, *anti* allyl C(H) *t*-Bu); 81.8 (s, *syn* allyl C(H) *t*-Bu); 35.6 (s, *t*-Bu CMe_3_) 34.5 (s, *t*-Bu CMe_3_); 31.1 (s, *t*-Bu (CH_3_)_3_); 30.6 (s, *t*-Bu (CH_3_)_3_). ^31^P{^1^H} NMR (162 MHz, C_6_D_6_, 298 K): δ 23.72 (Figures S7–S13 and S14 contains the spectrum of the product of the reaction mixture of [{A^2t^NiBr}_2_] with 2 equiv).

#### Synthesis of [A′Ni(PPh_3_)Br]

The same procedure detailed for [A^2t^Ni(PPh_3_)Br] was used here, substituting [{A′NiBr}_2_] for [{A^2t^NiBr}_2_]. The isolated product is a hard, dark red paste that is indefinitely stable in the glovebox atmosphere. Anal. calcd. for C_27_H_36_BrNiPSi_2_: C, 55.31; H, 6.19. Found: C, 55.74; H, 6.35. ^1^H NMR (600 MHz, d_8_-toluene, 298 K): δ 7.76 (irregular t, *J* = 8.7 Hz, 6H, PPh_3_ Ar–H); 7.03 (m, 9 H, PPh_3_ Ar–H); 5.69 (dd, *J* = 16.1, 9.1 Hz, 1 H, center allyl CH); 3.15 (dd, *J* = 16.1, 7.1 Hz, 1 H, allyl *anti* C(H) *t*-Bu); 2.76 (dd, *J* = 9.1, 5.7 Hz,1 H, allyl *syn* C(H) *t*-Bu); 0.50 (s, 9 H, TMS (CH_3_)_3_; 0.02 (s, 9 H, TMS (CH_3_)_3_). ^13^C{^1^H} NMR (151 MHz, toluene-*d*_8_, 298 K): δ 134.9 (d, *J* = 11.0 Hz, PPh_3_ C–H); 133.3 (d, *J* = 39.4 Hz, PPh_3_ ipso C); 130.2 (s, PPh_3_*para* C–H); 128.4 (d, *J* = 9.5 Hz, PPh_3_ C–H); 120.1 (s, allyl center C–H); 80.3 (d, *J* = 20.4 Hz, *anti* allyl C(H) *t*-Bu); 71.3 (d, *J* = 3.3 Hz, *syn* allyl C(H) *t*-Bu); 0.80 (s, TMS (CH_3_)_3_); 0.30 (s, TMS (CH_3_)_3_). ^31^P{^1^H} NMR (162 MHz, C_6_D_6_, 298 K): δ 23.60 (Figure S15).

#### Synthesis of [A^2t^Ni(IMes)Br] (**5**)

The same procedure detailed for [A^2t^Ni(PPh_3_)Br] was used here, substituting IMes for PPh_3_. [{A^2t^NiBr}_2_] (0.053 g, 0.082 mmol) and IMes (0.055 g, 0.18 mmol) were allowed to react in THF (5 mL) to yield 0.097 g (90% yield) of (**5**). The isolated product is a dark red, hard solid that is sparingly soluble in hexanes but soluble in aromatic hydrocarbons and polar organic solvents. The compound appears indefinitely stable in the solid state, but when stored in hexane solution, a white solid is slowly deposited. After removing the white solid, the NMR features of the compound were unchanged, making the decomposition mechanism unclear. A small amount of a second product is consistently present in NMR spectra, and its spectroscopic features are consistent with a *syn*, *syn* A^2t^ -nickel complex, but its exact nature is unknown. Due to its low concentration and broadened peaks, some of the signals belonging to this species cannot be found, but those observed are listed here. Anal. calcd. for C_32_H_45_BrN_2_Ni: C, 64.45; H, 7.61; N, 4.70. Found: C, 63.84; H, 6.67; N, 4.70. Combustion analysis was low in carbon and hydrogen; we refer the reader to see the provided NMR spectra in the SI as an additional measure of bulk purity. Major product: ^1^H NMR (400 MHz, C_6_D_6_, 298 K): δ 6.83 (s, 2H, Mes Ar–H); 6.81 (s, 2H, Mes Ar–H); 6.10 (s, 2 H, NHC HC = CH); 4.20 (dd, *J* = 14.6, 8.3 Hz, 1 H, center allyl CH); 3.27 (m, 2 H, allyl *syn* and *anti*-C(H) *t*-Bu); 2.29 (br s, 12 H, Ar–CH_3_); 2.25 (br s, 12 H, Ar–CH_3_); 2.14 (br s, 12 H, Ar–CH_3_); 1.24 (s, 9 H, *t*-Bu (CH_3_)_3_; 1.00 (s, 9 H, *t*-Bu (CH_3_)_3_). ^13^C{^1^H} NMR (101 MHz, C_6_D_6_, 298 K): δ 185.4 (s, NHC N–C–N); 138.9 (s, Mes *ipso* C); 137.0 (s, Mes *para* C-Me); 129.5 (s, Mes Ar–H); 129.5 (s, Mes Ar–H); 123.6 (s, NHC HC = CH); 98.8 (s, allyl center C–H); 91.6 (s, *anti* -allyl C(H)*t*-Bu); 68.9 (s, *syn*-allyl C(H)*t*-Bu); 34.5 (s, *t*-Bu CMe_3_); 33.9 (s, *t*-Bu CMe_3_); 32.0 (s, *t*-Bu (CH_3_)_3_); 30.3 (s, *t*-Bu (CH_3_)_3_). 21.2 (s, Ar–CH_3_); 19.8 (s, Ar–CH_3_). Minor product: ^1^H NMR (400 MHz, C_6_D_6_, 298 K): δ 6.79 (s, 4H, Mes Ar–H); 6.28 (br s, 2H, NHC HC = CH); 4.67 (t, *J* = 12.3 Hz, 1 H, center allyl CH); 2.36 (irregular br s, 14 H, Ar–CH_3_ and terminal allyl C–H); 2.08 (s, 6 H, Ar–CH_3_); 1.12 (s, 9 H, *t*-Bu (CH_3_)_3_. ^13^C{^1^H} NMR (101 MHz, C_6_D_6_, 298 K): 98.2 (s, allyl center C–H); 35.0 (s, *t*-Bu CMe_3_); 34.5 (s, *t*-Bu CMe_3_); 30.2 (s, *t*-Bu (CH_3_)_3_); 30.0 (s, *t*-Bu (CH_3_)_3_). 20.9 (s, Ar–CH_3_); 19.5 (s, Ar–CH_3_) (Figures S16, S17).

#### Synthesis of [A′Ni(IMes)Br] (**6**)

The same procedure detailed for [A^2t^Ni(PPh_3_)Br] was used here, substituting IMes for PPh_3_ and [{A′NiBr}_2_] for [{A^2t^NiBr}_2_]. [{A′NiBr}_2_] (0.011 g, 0.019 mmol) and IMes (0.012 g, 0.039 mmol) were allowed to react in THF (5 mL) to yield 0.023 g (quantitative) of (**6**). The isolated product is a hard, dark scarlet solid that is sparingly soluble in hexane and soluble in aromatic hydrocarbons and polar organic solvents. The compound appears indefinitely stable under N_2_. Anal. calcd. for C_30_H_45_BrN_2_NiSi_2_: C, 57.33; H, 7.22 N, 4.46. Found: C, 57.48; H, 7.36; N, 4.28. ^1^H NMR (400 MHz, C_6_D_6_, 298 K): δ 6.85 (s, 2H, Mes Ar–H); 6.81 (s, 2H, Mes Ar–H); 6.05 (s, 2 H, NHC HC = CH); 5.14 (dd, *J* = 15.9, 8.5 Hz, 1 H, center allyl CH); 2.95 (d, 1 H, allyl *syn*-C(H)TMS); 2.27 (br s, 6 H, Ar–CH_3_); 2.17 (s, 6 H, Ar–CH_3_); 2.17 (d, 15.9 Hz, 1 H, *anti*-allyl C(H)TMS); 2.16 (br s, 6 H, Ar–CH_3_); 0.26 (s, 9 H, TMS (CH_3_)_3_; 0.05 (s, 9 H, TMS (CH_3_)_3_). ^13^C{^1^H} NMR (151 MHz, C_6_D_6_, 298 K): δ 182.6 (s, NHC N–C–N); 139.0 (s, Mes *ipso* C); 136.8 (s, Mes *para* C-Me); 136.2 (s, Mes *ortho* C-Me); 129.7 (s, Mes Ar–H); 129.5 (s, Mes Ar–H); 123.6 (s, NHC HC = CH); 118.2 (s, allyl center C–H); 73.1 (s, *anti*-allyl C(H)TMS); 59.7 (s, *syn*-allyl C(H)TMS); 21.0 (s, Ar–CH_3_); 19.7 (s, Ar–CH_3_); 19.2 (s, Ar–CH_3_); 1.2 (s, TMS (CH_3_)_3_); 0.0 (s, TMS (CH_3_)_3_) (Figures S18).

### General Procedures for X-ray Crystallography

All crystals used for crystallographic analysis were grown by slow evaporation from hexane solution in glass vials. A suitable crystal of each sample was selected for analysis and mounted in a polyimide loop. All measurements were made on a Rigaku Oxford Diffraction Supernova Eos CCD with Cu–Kα or Mo–Kα radiation at a temperature of 100 K (or 294 K for (**7**)). Using Olex2,^97^ the structures were solved with the ShelXT^98^ structure solution program using Direct Methods and refined with the ShelXL refinement package^99^ using least-squares minimization. Prof. Nathan Schley (Vanderbilt Univ.) performed the data collection and structure solution for (**2**), (**3**), and (**4**); Prof. William Brennessel (Univ. of Rochester) did the same for (**5**), (**6**), and (**7**).

### General Procedures for Calculations

All calculations were performed with the Gaussian 16 (Linux) suite of programs.^100^ The B3PW91 functional, which incorporates Becke’s three-parameter exchange functional^101^ with the 1991 gradient-corrected correlation functional of Perdew and Wang,^102^ was used. For dispersion-corrected calculations, Grimme’s D3 correction^103^ with additional Becke-Johnson damping was used^104^ (Gaussian keyword: empiricaldispersion = GD3BJ). For the energy of deprotonation (HA → [A]^−^ + H^+^), the def2-TZVPD basis set was used on all atoms. For other calculations, the def2-TZVP basis was used for all atoms.^105^ For all calculated CO stretching frequencies, the PW91PW91 functional was used; its combination with the def2-TZVP basis has been shown to generate reliable frequencies, even without using a scaling factor.^106^ ETS-NOCV (Extended Transition State–Natural Orbitals for Chemical Valence) fragmentation analysis was calculated with the Multiwfn program.^95^

## Data Availability

The data underlying this study are available in the published article and its Supporting Information.

## Supporting Information Available

NMR spectra, computational, and crystallographic details (PDF)
Optimized coordinates of structures (XYZ)

## Supplementary Material

gg4c00044_si_001.pdf

gg4c00044_si_002.xyz
