# Supplementary material for: t-Butyl and Trimethylsilyl Substituents in Nickel Allyl Complexes: Similar but Not the Same
Source: ACS Org Inorg Au. 2024 Sep 17;4(6):658–72. doi: 10.1021/acsorginorgau.4c00044 (PMC11621960; doi:10.1021/acsorginorgau.4c00044)
Supplement: Supplementary file 1 — gg4c00044_si_001.pdf [file gg4c00044_si_001.pdf]

SUPPORTING INFORMATION

FOR

t-Butyl and trimethylsilyl substituents in nickel allyl complexes: similar but not the same

Henry P. DeGroot,[a] Isaiah R. Speight,[a] William W. Brennessel,[b] and Timothy P. Hanusa*[a]

[a]Department of Chemistry, Vanderbilt University, PO Box 1822, Nashville, TN 37235 (USA)

[b]X-ray Crystallographic Facility, Department of Chemistry, University of Rochester, Rochester, NY 14627 (USA)

Figures S1–S18: ^{1}H, ^{13}C, and ^{31}P NMR Spectra S2

Table S1: Calculated relative energies of selected A^{2t}-nickel complexes S16

Table S2: Energetics of Formation of $[A^{2t}Ni(PPh_3)Br]$ S17

Figure S19: Thermal ellipsoid plot of 5-hydroxy-2,2,6,6-tetramethylheptan-3-yl pivalate (**3**) ... S18

Figure S20: Orbital interaction in $[A'NiBrPPh_3]$........ S19

Table S3: Crystal Data and Summary of X–ray Data Collection S20

References S29

Figure S1. ^{1}H NMR (400 MHz) of *anti*-5-hydroxy-2,2,6,6-tetramethylheptan-3-yl pivalate (**3**) at 298 K in $CDCl_3$. Peaks belonging to 2,2,6,6-tetramethylhept-4-en-3-one are marked with an asterisk.

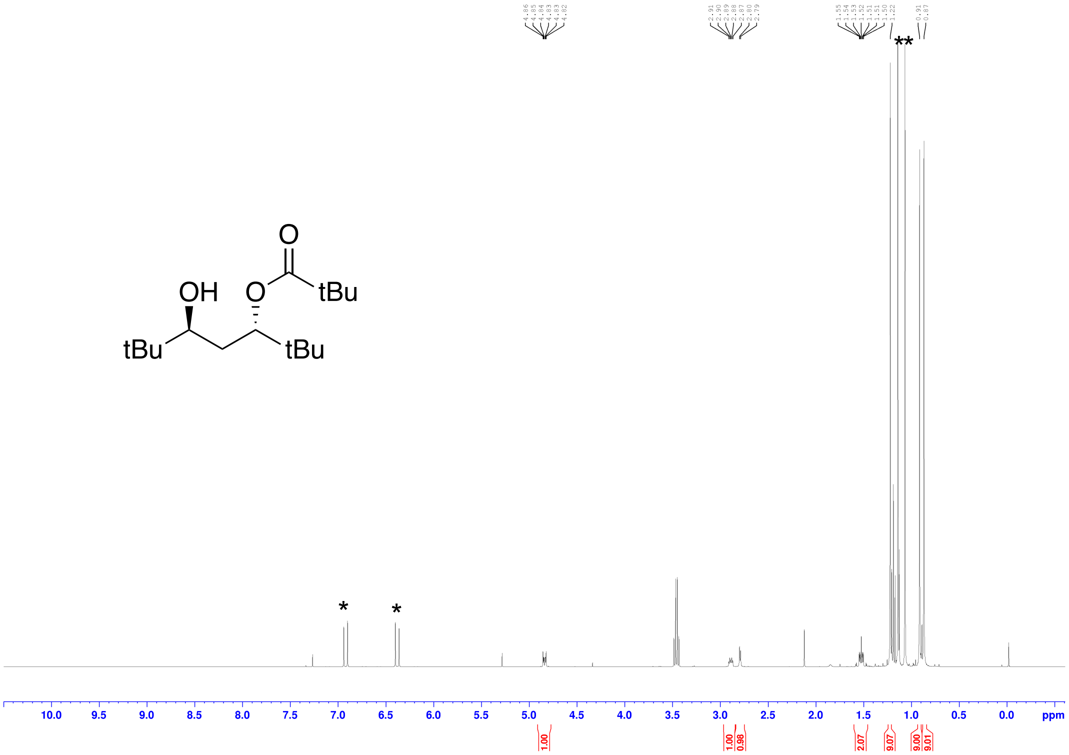

Figure S2. ^{1}H NMR (400 MHz) of 5-(*tert*-butyl)-2,2,8,8-tetramethylnonane-3,7-dione (**2**) in $CDCl_3$

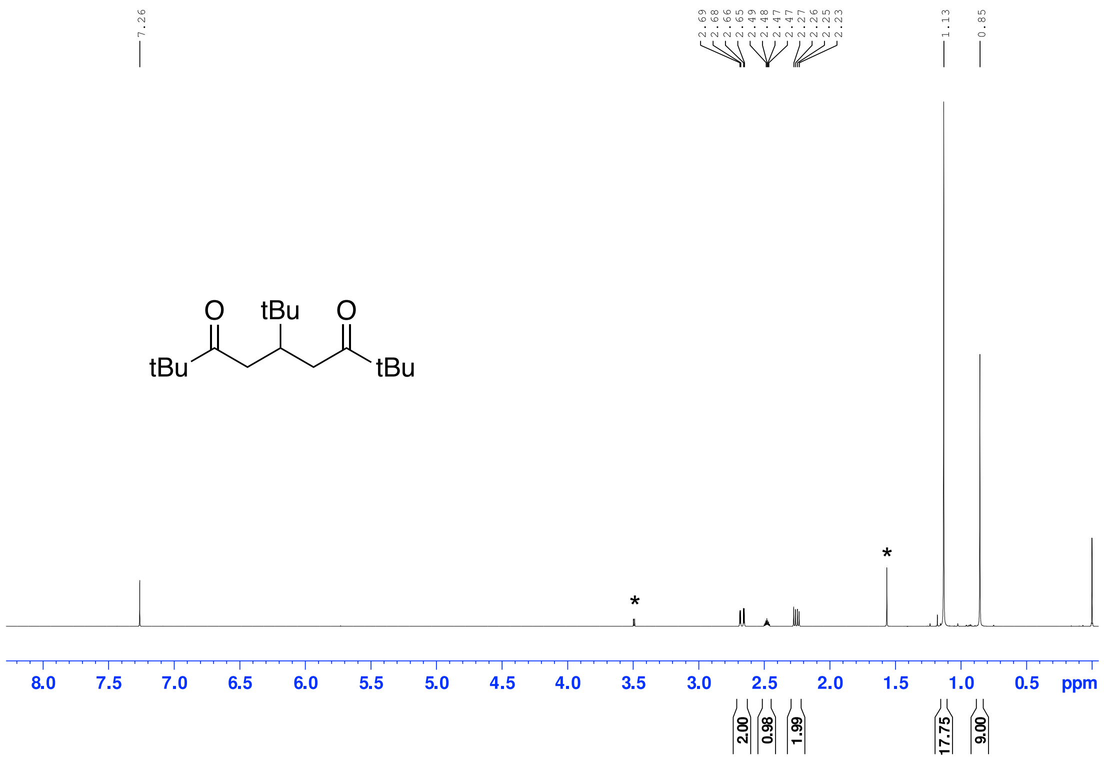

Figure S3. $^{13}C\{^{1}H\}$ NMR (101 MHz) of 5-(*tert*-butyl)-2,2,8,8-tetramethylnonane-3,7-dione (**2**) in $CDCl_3$

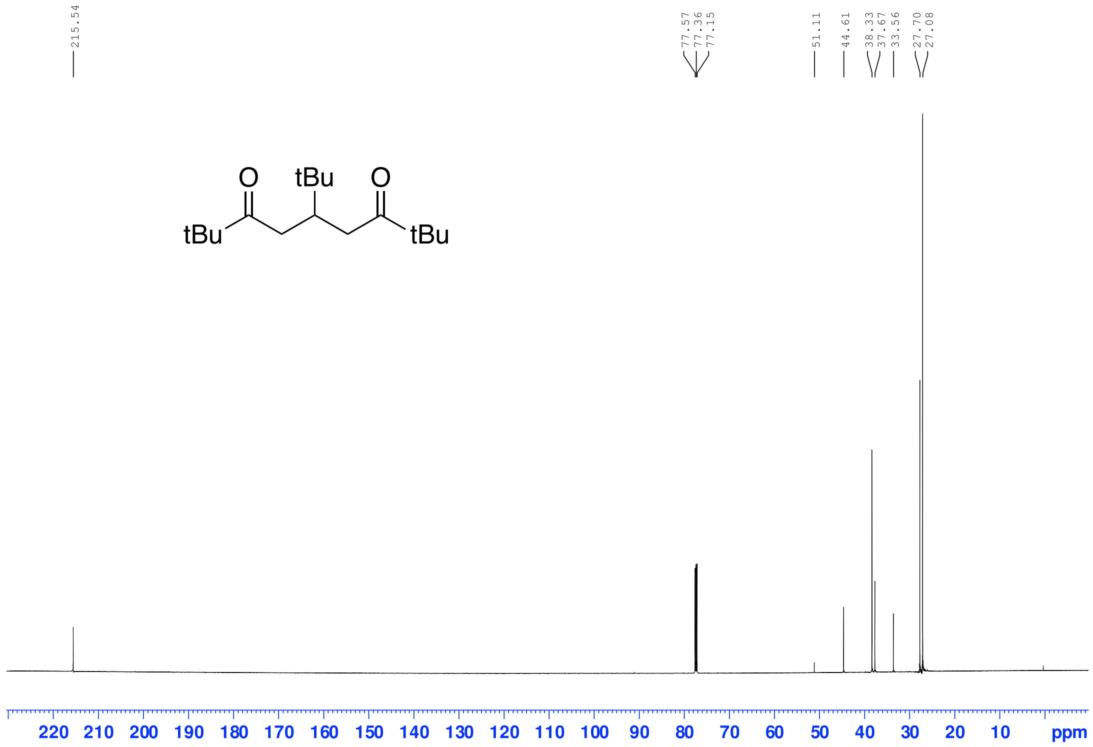

Figure S4. ^{1}H NMR (400 MHz) of [{A^{2t}NiBr}$_2$] at 298 K in C_6D_6. The low solubility of the compound in arenes is reflected in the corresponding greater intensities of normally trace impurities (e.g., grease).

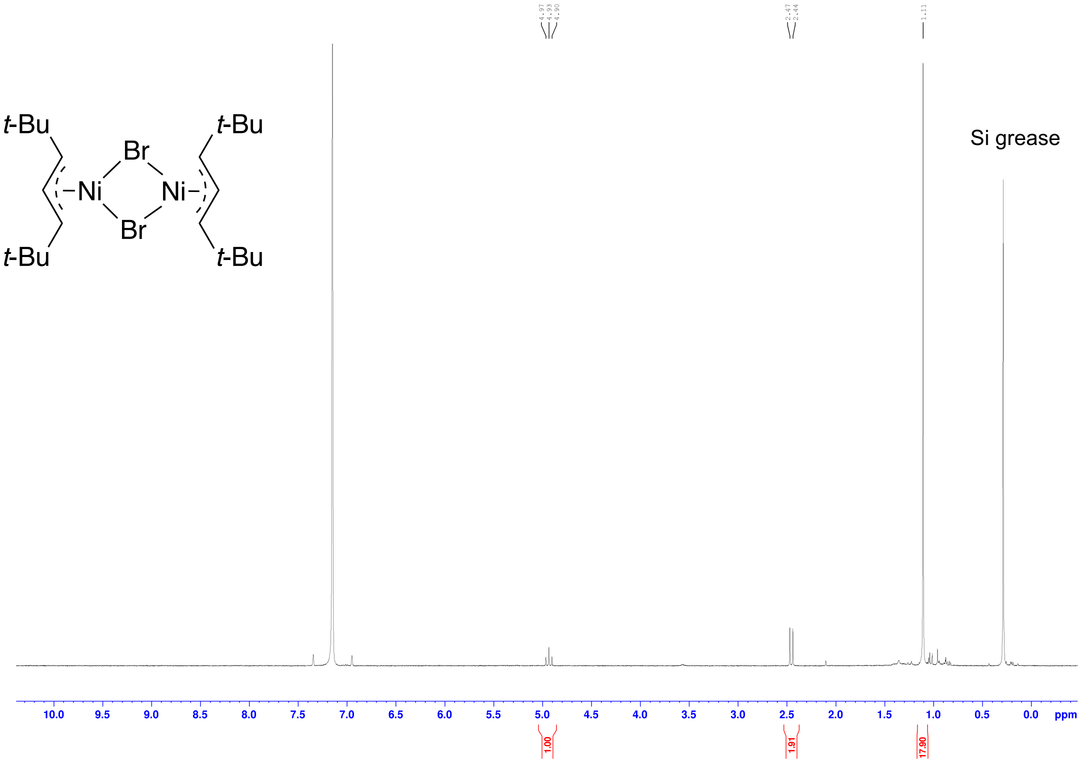

Figure S5. ^{13}C{^{1}H} NMR (151 MHz) of [{A^{2t}NiBr}$_2$] in C_6D_6

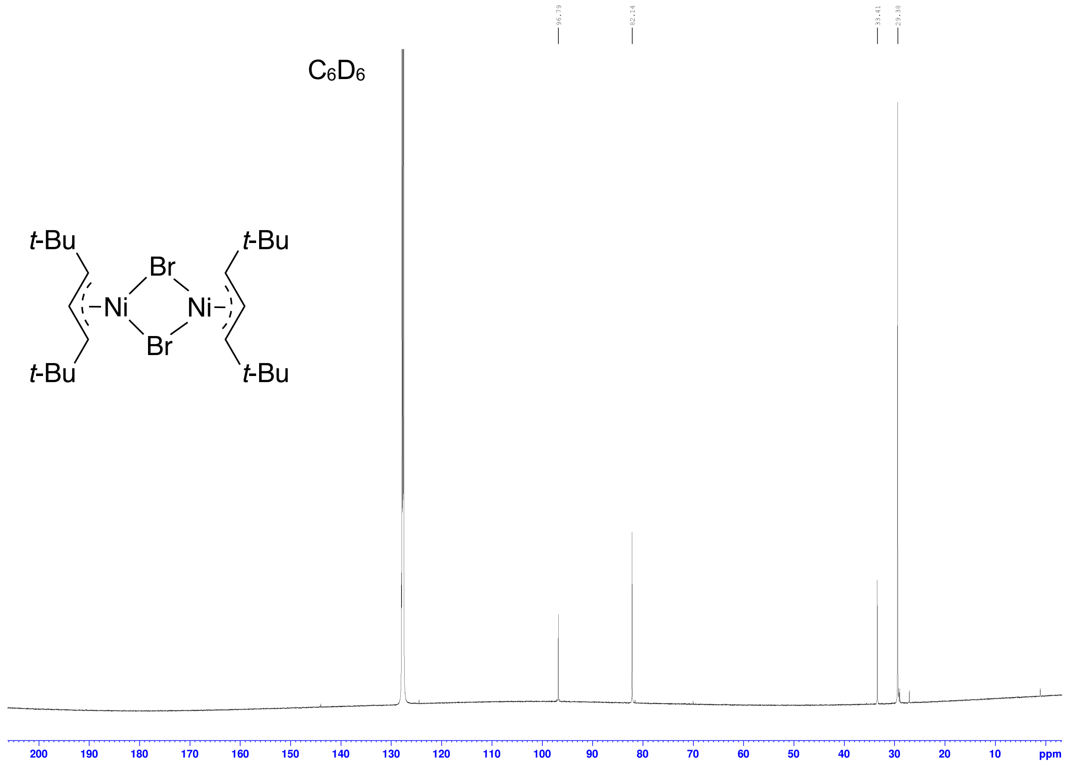

Figure S6. ^{1}H NMR (400 MHz) of [{A´NiBr}$_2$] in C_6D_6, including an enlargement of the TMS region. Peaks belonging to the previously known *syn*, *syn* product are marked with S/S.

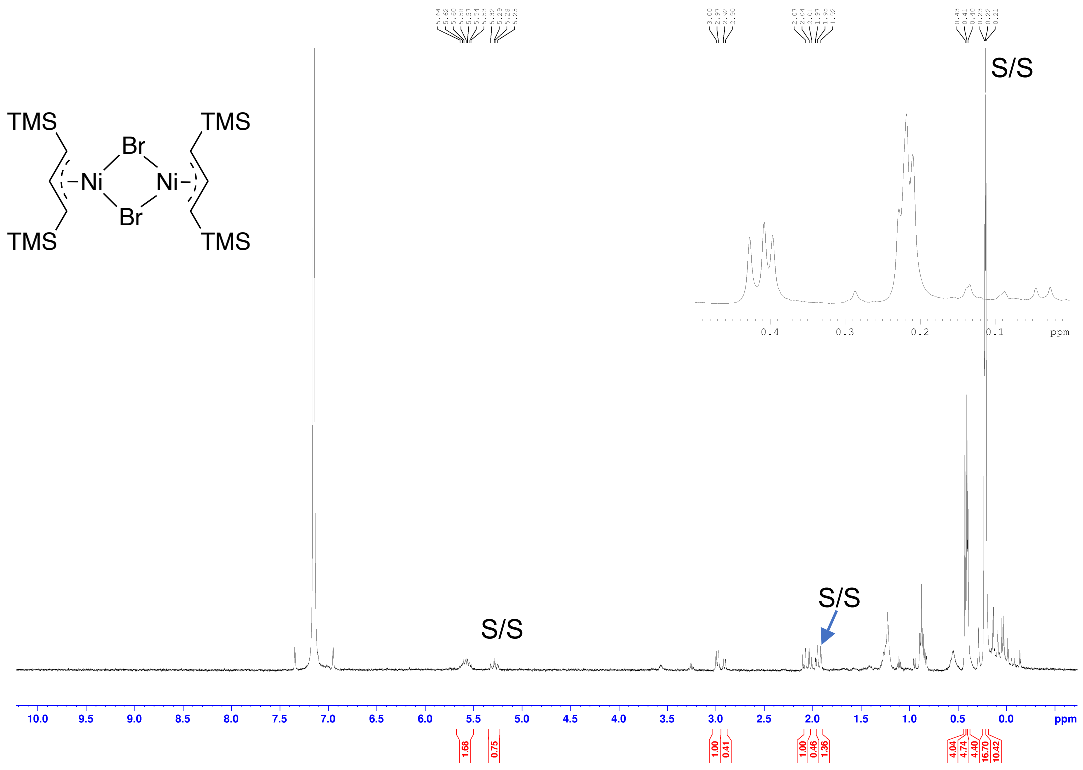

Figure S7. ^{1}H NMR (600 MHz) of [$A^{2t}Ni(PPh_3)Br$] in toluene-d_8 at room temperature; peaks belonging to [{$A^{2t}NiBr$}$_2$], free PPh_3, or toluene are marked with an asterisk, while peaks where [$A^{2t}Ni(PPh_3)Br$] overlaps with another species are marked with an O. The peak marked I represents an impurity in the toluene-d_8, present in the majority of spectra taken in d_8-toluene across multiple batches from the same supplier.

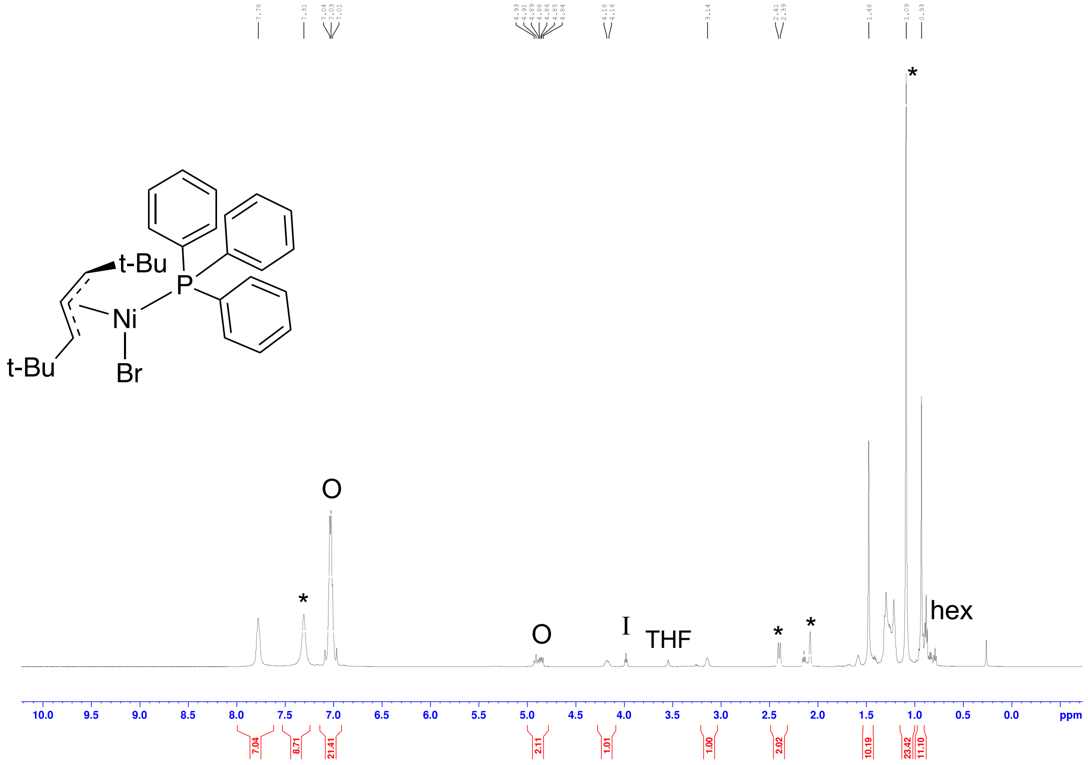

Figure S8. ^{1}H-^{1}H COSY NMR (400 MHz) of [$A^{2t}Ni(PPh_3)Br$] in C_6D_6 at 298 K. Allylic proton couplings are marked with an A.

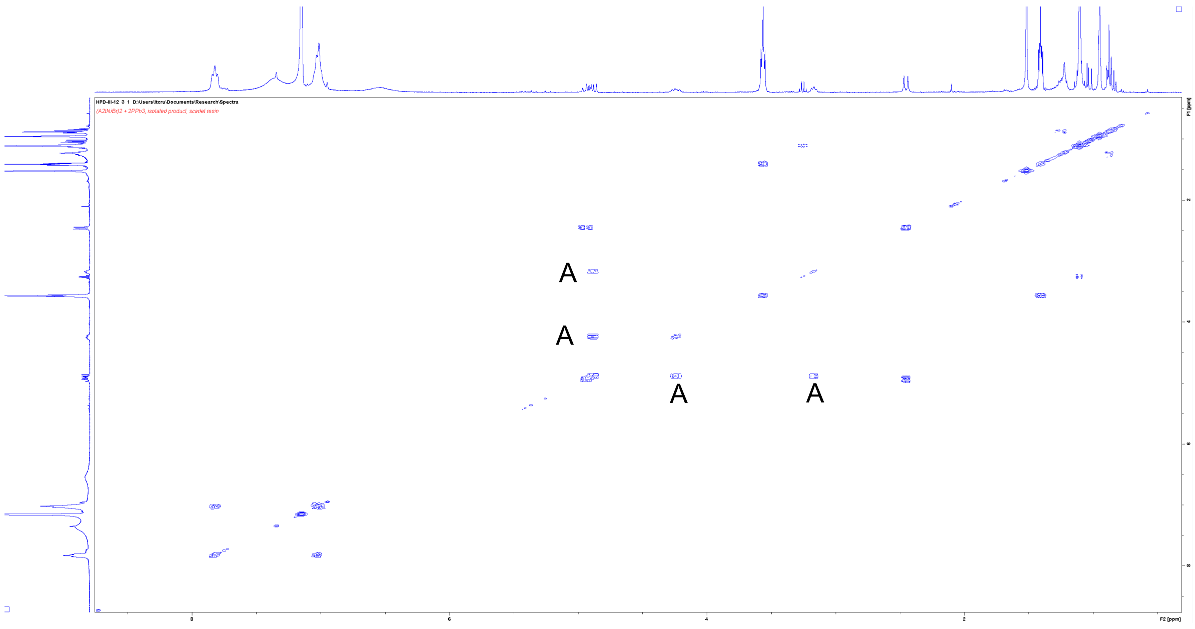

Figure S9. ^{1}H NMR (600 MHz) of [$A^{2t}Ni(PPh_3)Br$] in toluene-d_8; although spectra were measured at 258, 268, 278, and 288 K, for clarity only the spectra at 258 K and 288 K are shown. Peaks belonging to [$\{A^{2t}NiBr\}_2$] are marked with an asterisk, while peaks belonging to [$A^{2t}Ni(PPh_3)Br$] are marked with a P. The peak marked I represents a likely intermediate for PPh_3 exchange (see the main text).

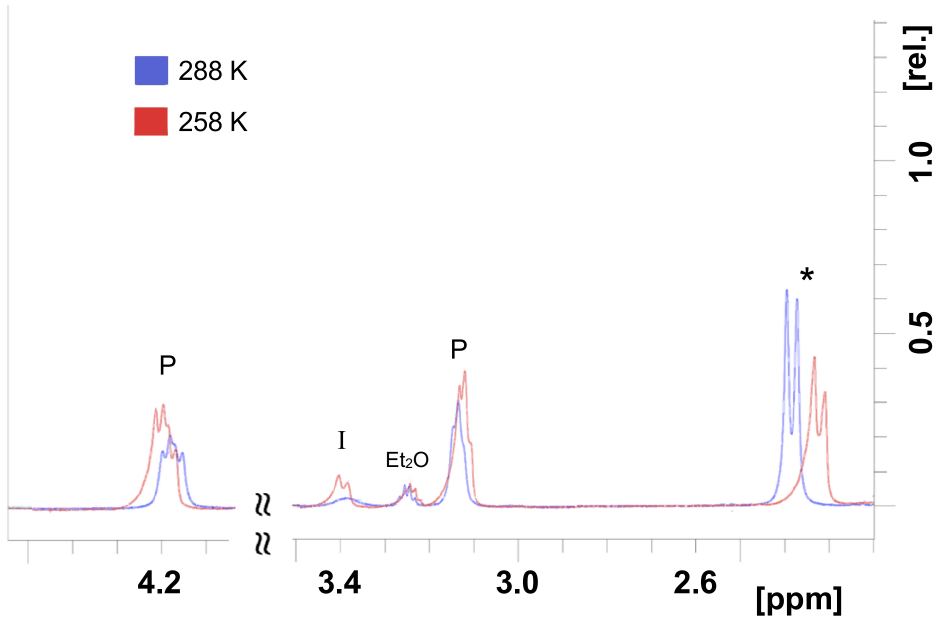

Figure S10. ^{1}H NMR (600 MHz) of [A^{2t}Ni(PPh$_3$)Br] in toluene-d$_8$ at 253 K; peaks belonging to [{A^{2t}NiBr}$_2$], free PPh$_3$, or toluene are marked with an asterisk, while peaks where [A^{2t}Ni(PPh$_3$)Br] overlaps with another species are marked with an O. Phasing was done to optimize a consistent base-line for the purposes of integration; with other phasing parameters, evenly distributed peaks can be achieved. The peak marked I represents a consistent impurity in the d$_8$-toluene

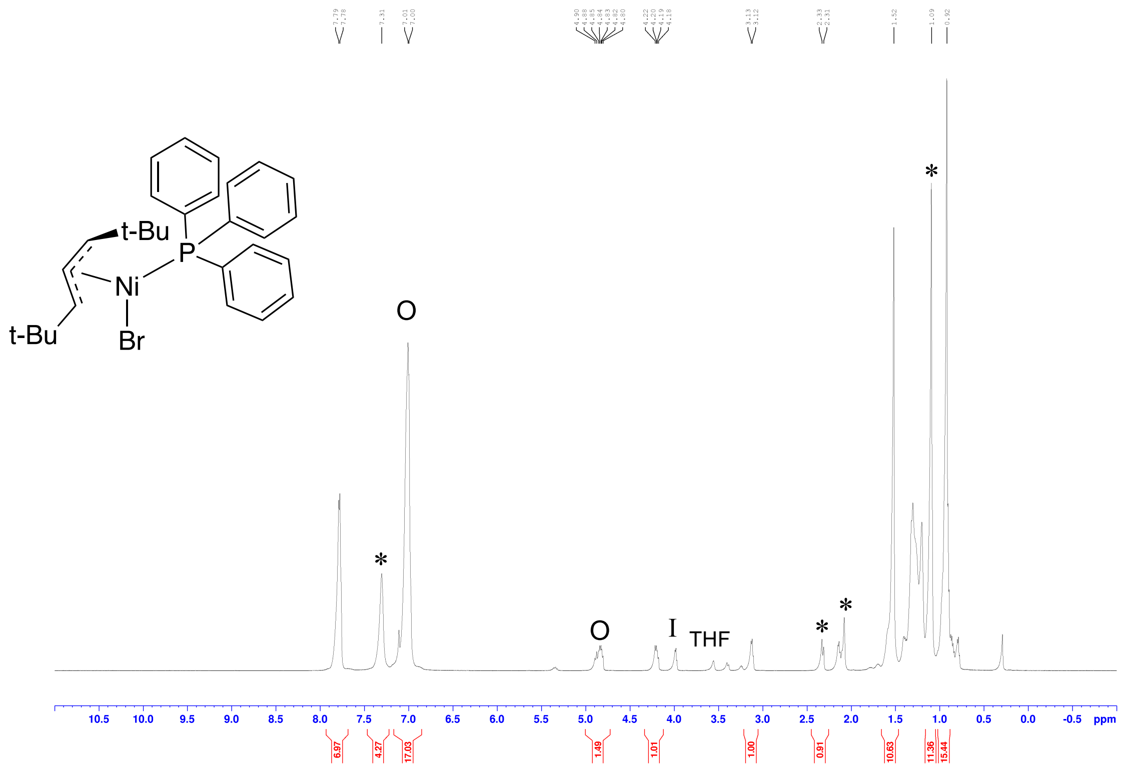

Figure S11· $^{13}C\{^{1}H\}$ NMR (151 MHz) of [$A^{2t}Ni(PPh_3)Br$] in toluene-d_8 at room temperature; peaks belonging to [$\{NiBr(A^{2t})\}_2$] or are marked with an asterisk, while peaks where [$A^{2t}Ni(PPh_3)Br$] overlaps with another species are marked with an O.

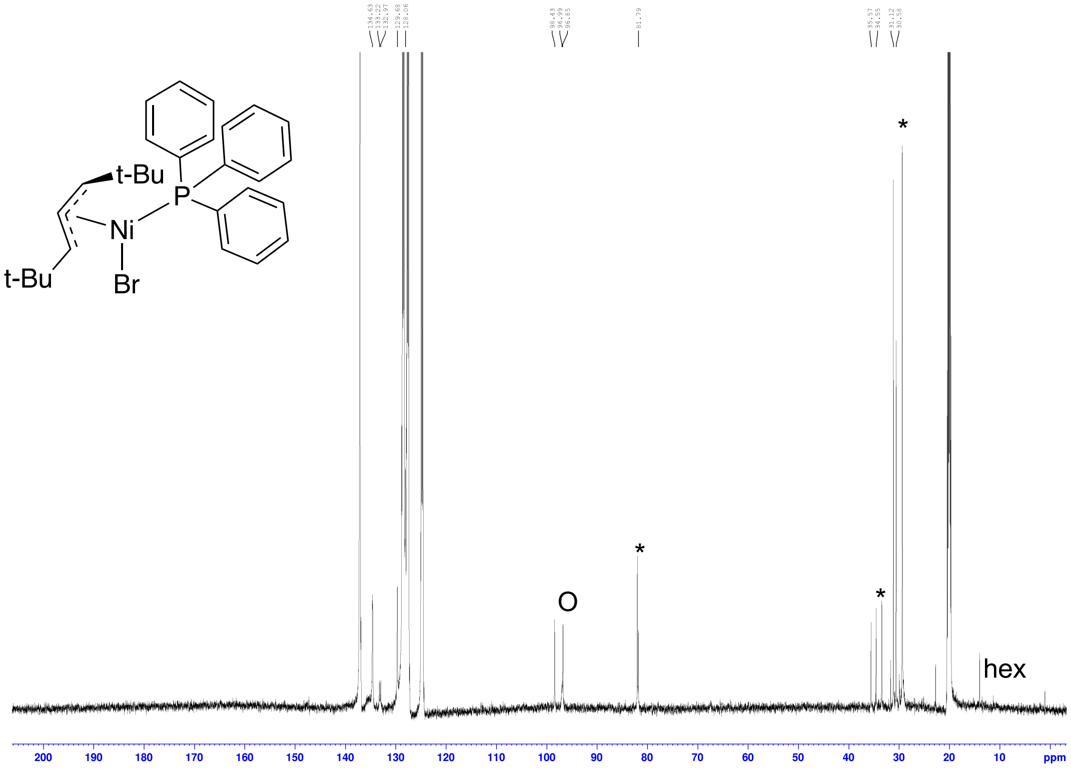

Figure S12. ^{1}H-^{13}C HSQC NMR (600-151 MHz) of [$A^{2t}Ni(PPh_3)Br$] in toluene-d_8 at room temperature. Allylic proton-carbon couplings are marked with an A.

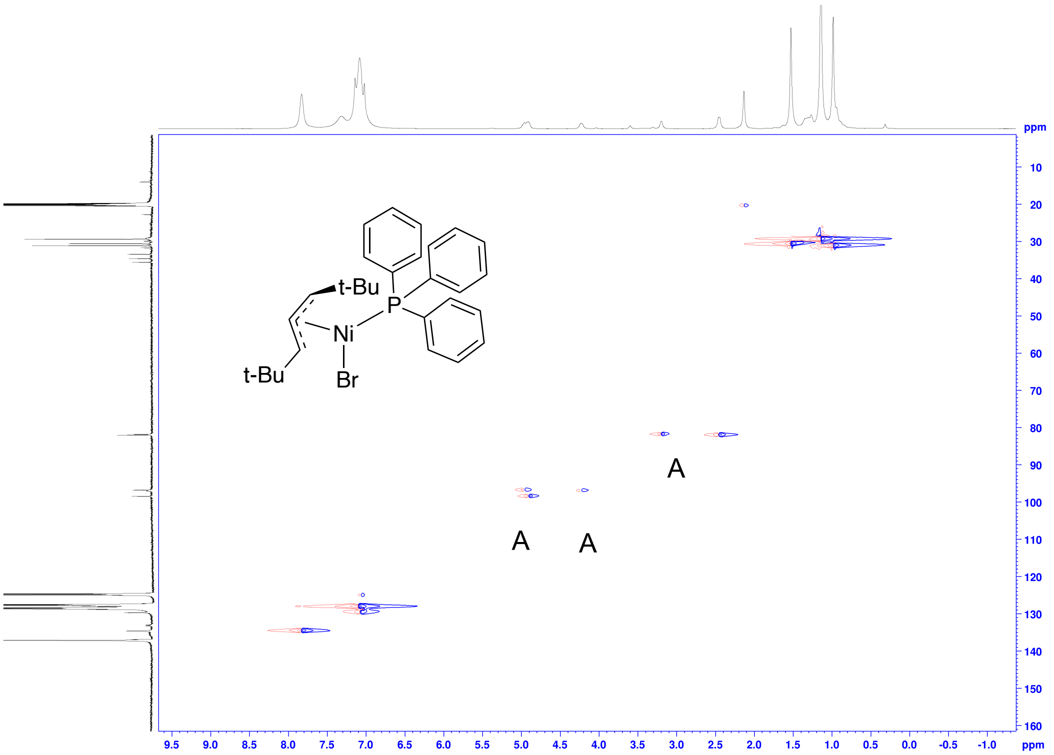

Figure S13. $^{31}P\{^{1}H\}$ NMR (162 MHz) of [$A^{2t}Ni(PPh_3)Br$] in C_6D_6 at room temperature.

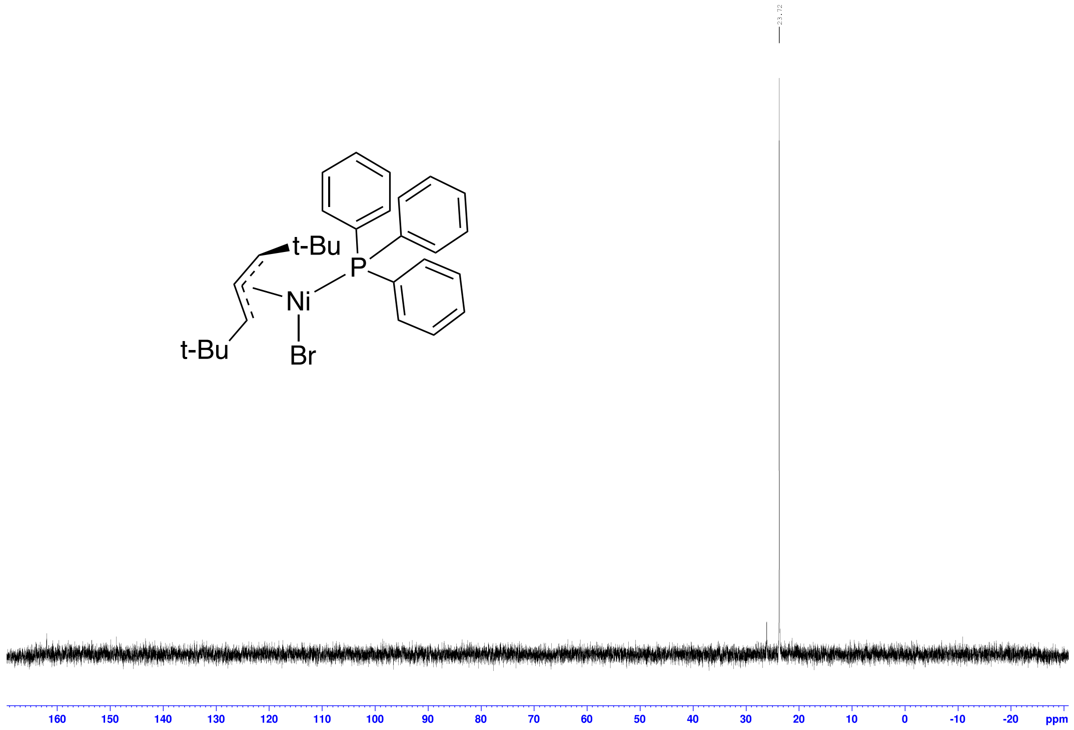

Figure S14. ^1H NMR (400 MHz) of reaction mixture of [{$A^{2t}NiBr$}$_2$] with 2 equivalents of tri-*n*-butylphosphine in C_6D_6 at room temperature. The presence of only one species whose spectral features are similar to [$A^{2t}Ni(PPh_3)Br$] strongly suggests quantitative formation of an adduct of the form [$A^{2t}Ni(P^nBu_3)Br$]. The low solubility of the compound in C_6D_6 is reflected in the corresponding greater intensities of normally trace impurities.

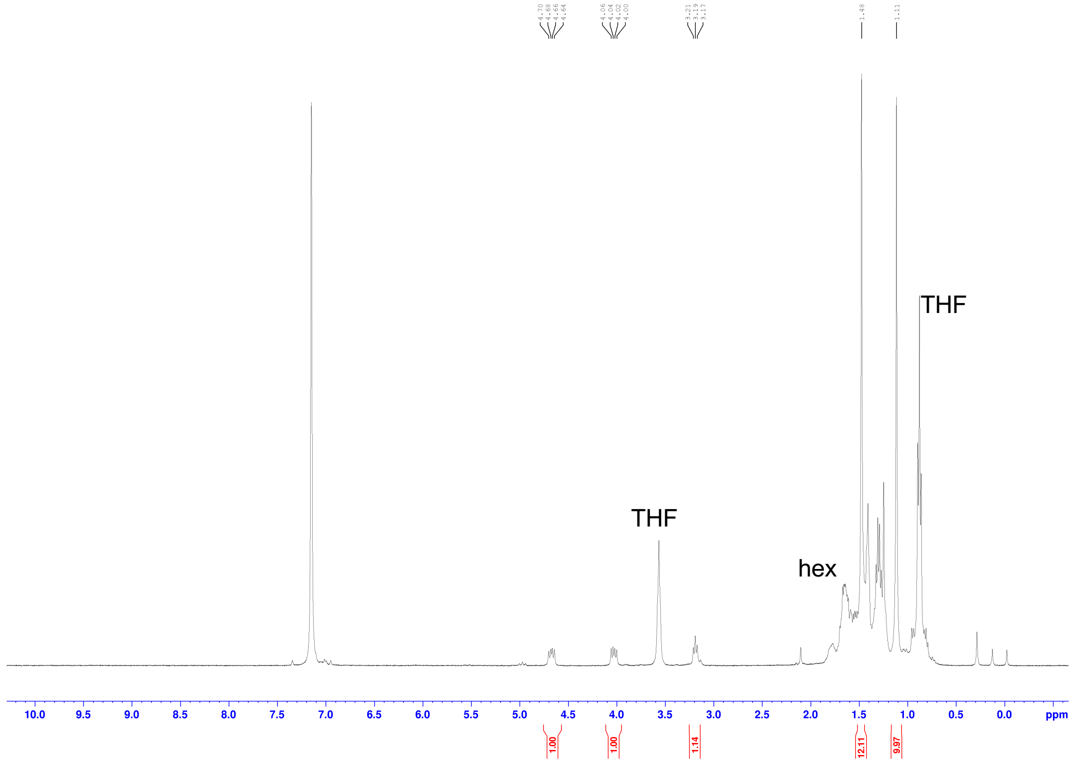

Figure S15. ^{1}H-^{13}C HSQC NMR (600-151 MHz) of [A´Ni(PPh3)Br] in toluene-d_8 at room temperature.

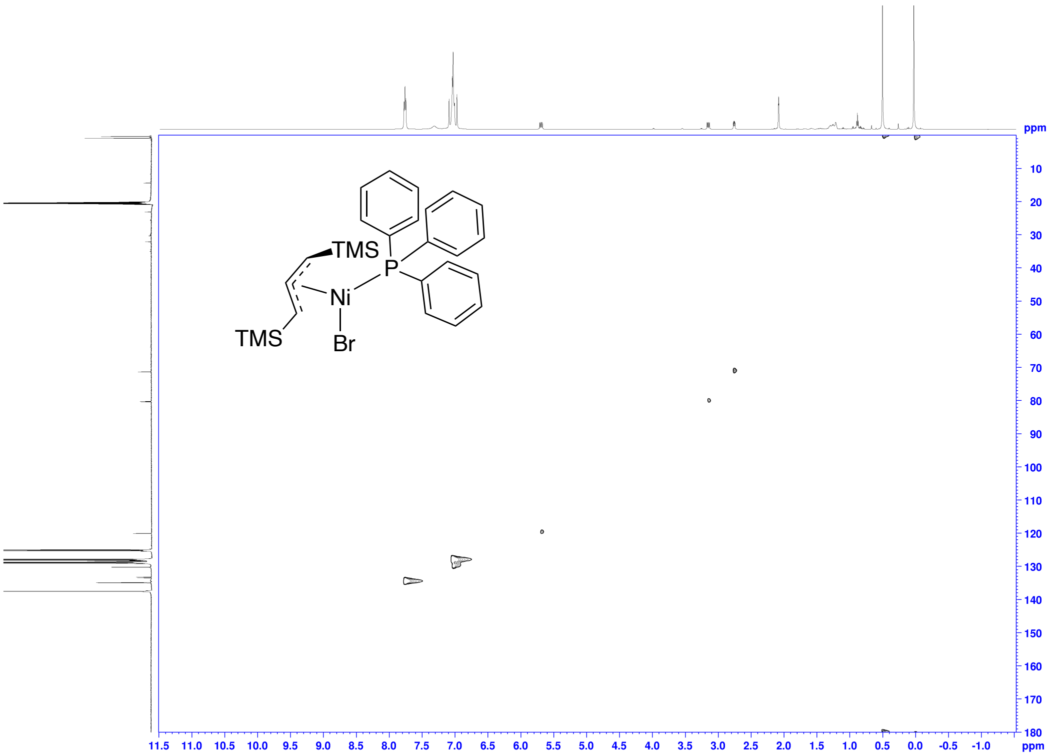

Figure S16. ^{1}H NMR (400 MHz) of [A^{2t}Ni(IMes)Br] (**5**) in C_6D_6 at room temperature. Peaks belonging to the minor product are marked with an M.

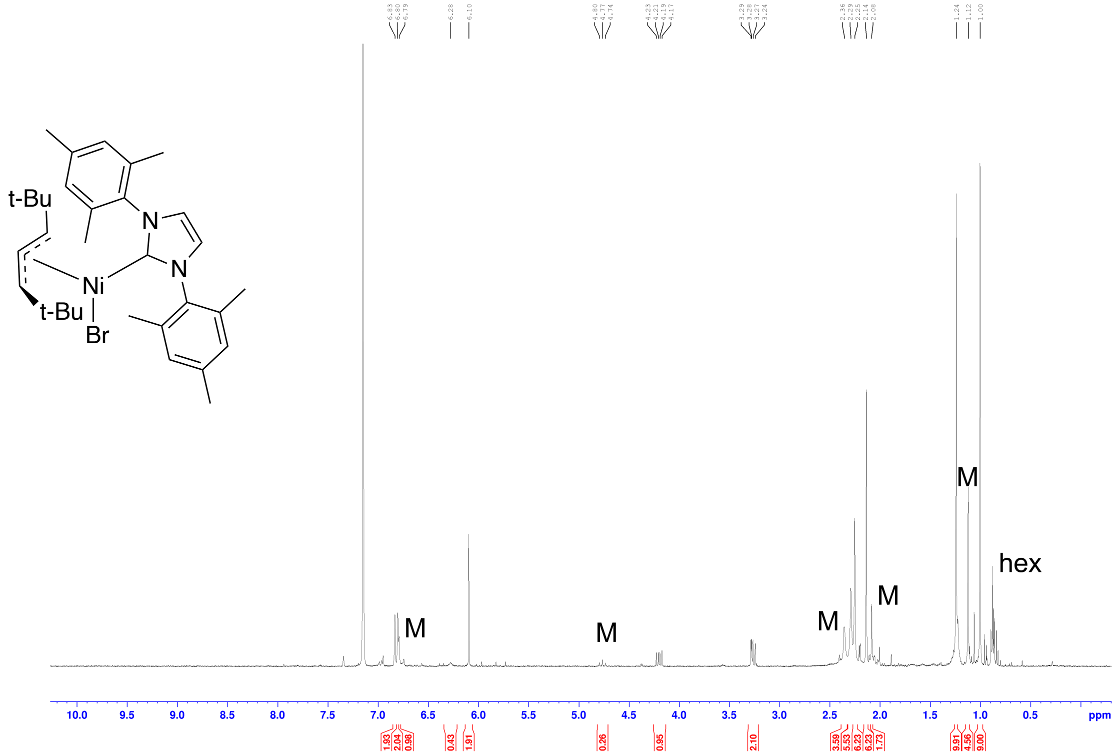

Figure S17. ^{1}H-^{13}C HSQC NMR (400-101 MHz) of [A^{2t}Ni(IMes)Br] in C_6D_6 at 298 K. Allylic proton-carbon couplings are marked with an A.

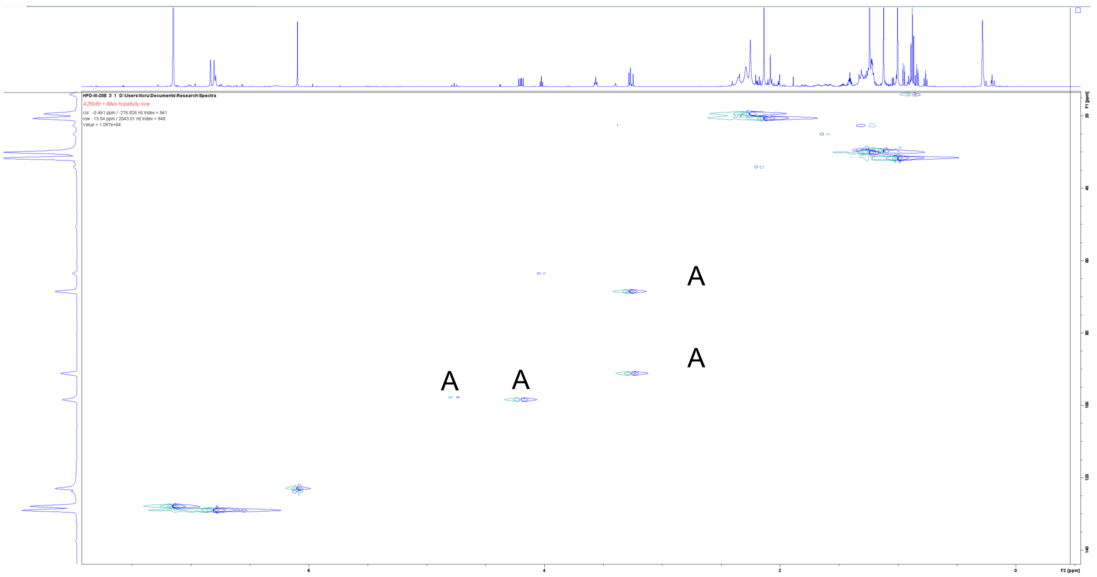

Figure S18. ^{1}H-^{13}C HSQC NMR (600-151 MHz) of [A´Ni(IMes)Br] (**6**) in C_6D_6 at 298 K. Allylic proton-carbon couplings are marked with an A.

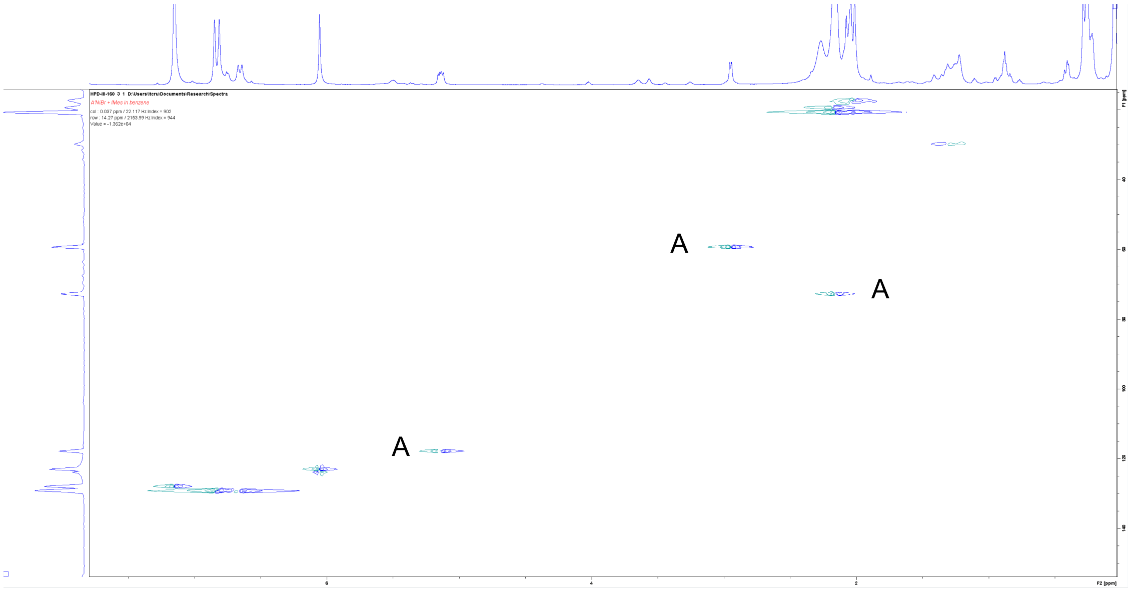

Table S1: Calculated relative energies (B3PW91-D3BJ/def2TZVP) of configurations of selected A^{2t}-nickel complexes

[{A^{2t}NiBr}$_2$]

Isomer	Energy (ΔG°, Hartrees)	Rel. Energy (ΔG°, kJ mol^{-1})
Eclipsed *syn*,*syn*/*syn*,*syn*	-9028.454539	0.00
Eclipsed *syn*,*syn*/*syn*,*anti*	-9028.449938	12.08
Eclipsed *syn*,*anti* /*syn*,*anti* (trans)	-9028.446737	20.48
Eclipsed *syn*,*anti* /*syn*,*anti* (cis)	-9028.444546	26.24
Eclipsed *anti*,*anti* /*anti*,*anti*	-9028.395249	155.67
Staggered *syn*,*syn*/*syn*,*syn*	-9028.450845	9.70
Staggered *syn*,*syn*/*syn*,*anti*	-9028.450001	11.91
Staggered *syn*,*anti* /*syn*,*anti* (cis)	-9028.442375	31.94

[A^{2t}Ni(PPh_3)Br]

Isomer	Energy (ΔG°, Hartrees)	Rel. Energy (ΔG°, kJ mol^{-1})
syn,*syn*	-5550.345462	5.99
syn,*anti* (P trans to *syn t*-Bu)	-5550.347744	0.00
syn,*anti* (P trans to *anti t*-Bu)	-5550.341071	17.52

[A^{2t}Ni(IMes)Br]

Isomer	Energy (ΔG°, Hartrees)	Rel. Energy (ΔG°, kJ mol^{-1})
syn,syn	-5438.157555	5.24
syn,anti (carbene trans to syn t-Bu)	-5438.165912	0.00

Formation of [$A^{2t}Ni(PPh_3)Br$]

The ratio of the monomeric adduct and bromide bridged dimer at various temperatures was observed by variable temperature NMR in d^8-toluene solution. It was then endeavored to calculate the apparent equilibrium constant for this reaction at each temperature. For these purposes, the reaction has been formally written according to the following equation:

$$1\ [\{A^{2t}NiBr\}_2]\ +\ 2\ PPh_3 \rightarrow 2\ [A^{2t}Ni(PPh_3)Br]$$

This equation has an equilibrium constant with the following expression:

$$K = \frac{[[A^{2t}Ni(PPh_3)Br]]^2}{[[\{A^{2t}NiBr\}_2]] \times [PPh_3]^2}$$

Given that the ratio of [$\{NiBr(A^{2t})\}_2$] to free PPh_3 should remain at a constant value of 1 : 2 at all times, the equilibrium constant can then be written purely in terms of the two nickel complexes, yielding the following expression:

$$K = \frac{[[A^{2t}Ni(PPh_3)Br]]^2}{4 \times [[\{A^{2t}NiBr\}_2]]^3}$$

The NMR spectra have been calibrated to [$A^{2t}Ni(PPh_3)Br$] so that its concentration is always set to unity. Under this system, a single-term expression for the equilibrium constant is achieved, into which the apparent integration value for [$\{NiBr(A^{2t})\}_2$] in each expression can be inserted. The upfield doublet of [$\{NiBr(A^{2t})\}_2$] divided by four was used as its integration value in all cases. The calculated value of the equilibrium constant was then used to calculate the associated Gibbs free energy at each temperature.

Table S2: NMR ratio of [$A^{2t}Ni(PPh_3)Br$] to [$\{A^{2t}NiBr\}_2$]

Temp (K)	[$A^{2t}Ni(PPh_3)Br$] : [$\{A^{2t}NiBr\}_2$]	Calc. K	Calc. ΔG (kJ mol^{-1})
298	2.00	2.00	-1.72
288	2.38	3.37	-2.91
278	2.86	5.83	-4.08
268	3.44	10.3	-5.19
258	4.17	18.1	-6.21
253	4.35	20.5	-6.36

From the relationship ln K = $^{-\Delta H^\circ}/_{RT}$ + $^{\Delta S^\circ}/_{R}$, a plot of 1/T vs ln K (at right) allows extraction of ΔH° = -33.25 kJ mol^{-1} and ΔS° = -105.3 J mol^{-1}

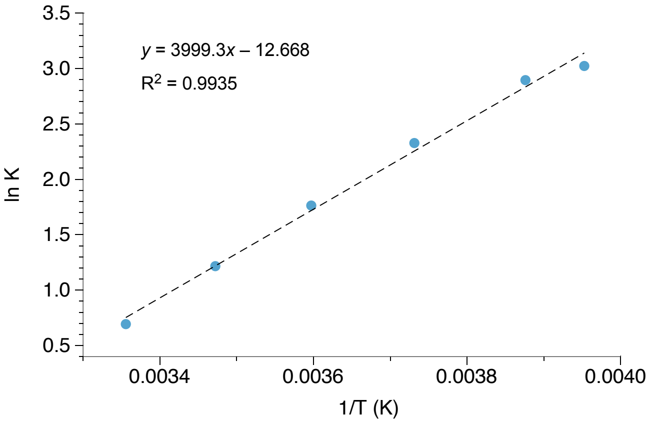

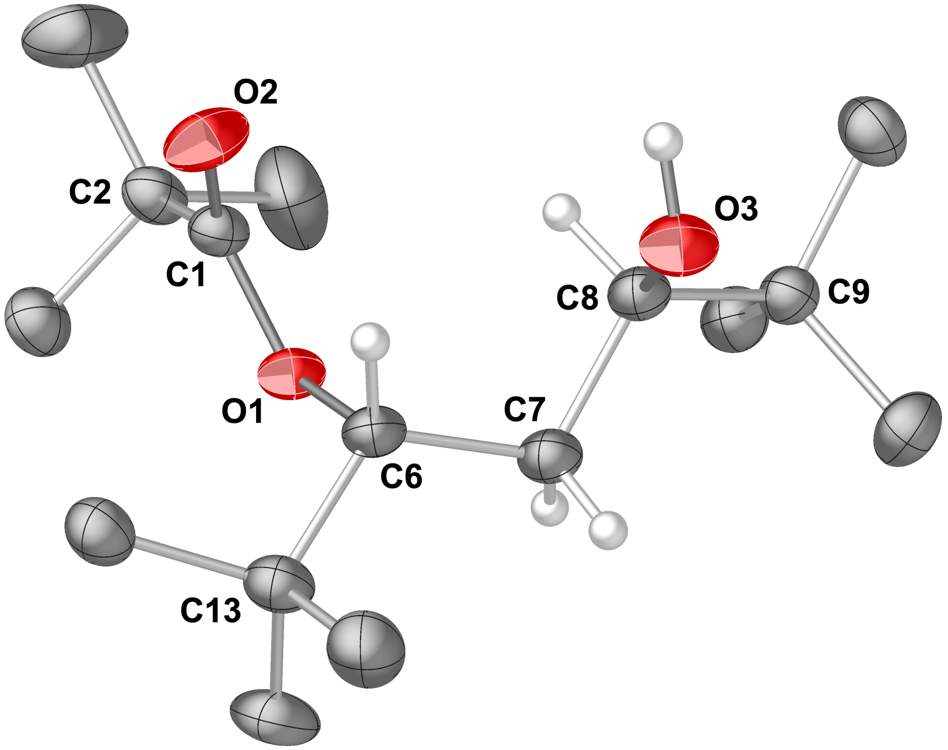

Figure S19. Thermal ellipsoid plot (50% level) of 5-hydroxy-2,2,6,6-tetramethylheptan-3-yl pivalate (**3**); for clarity, hydrogen atoms have been removed from the *t*-Bu groups, and the rest have been given arbitrary radii. Selected bond distances (Å) and angles (°): O1–C1, 1.3378(16); O1–C6, 1.4680(15); O2–C1, 1.2097(17); O3–C8, 1.4303(16); C1–C2, 1.5222(19); C6–C7, 1.5188(18); C6–C13, 1.5403(18); C7–C8, 1.5259(18); C8–C9, 1.5420(18); C1–O1–C6, 119.92(10); O1–C1–C2, 111.45(11); O2–C1–O1, 123.99(13); O2 C1 C2, 124.55(12); O1–C6–C7, 104.70(10); O1–C6–C13, 108.11(10); C7–C6–C13, 117.55(11); C6–C7–C8, 110.68(10); O3–C8–C7, 105.78(11); O3–C8–C9, 111.85(11); C7–C8–C9, 115.21(11).

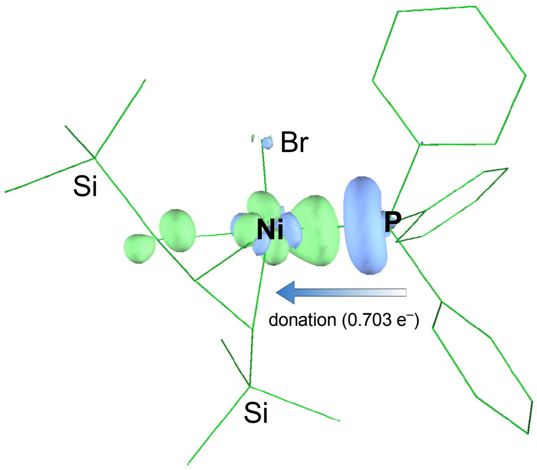

A′NiBrPPh$_3$

Figure S20. Blue and green isosurfaces display the region where the electron density decreases and increases, respectively, owing to the orbital interaction described by NOCV pair 1 (ΔE_1^{orb}) in [A´NiBrPPh3].

Table S3: Crystal Data and Summary of X–ray Data Collection

	5-(*t*-Bu)-2,2,8,8-tetramethylnonane-3,7-dione (2)	5-hydroxy-2,2,6,6-tetramethyl-heptan-3-yl pivalate (3)	[{A^{2t}NiBr}$_2$] (4)	[A^{2t}Ni(IMes)Br] (5)	[A´Ni(IMes)Br] (6)
Empirical formula	$C_{17}H_{32}O_2$	$C_{16}H_{32}O_3$	$C_{22}H_{42}Br_2Ni_2$	$C_{32}H_{45}BrN_2Ni$	$C_{30}H_{45}BrN_2NiSi_2$
Formula weight	268.42	272.41	583.79	596.32	628.48
Temperature	100.00(10) K	99.99(10) K	100.00(10) K	100.00(10) K	100.00(10) K
Wavelength	1.54184 Å	1.54184 Å	1.54184 Å	1.54184 Å	0.71073 Å
Crystal system	Monoclinic	Triclinic	Triclinic	Triclinic	Monoclinic
Space group	$P2_1/c$	P-1	P-1	P-1	$P2_1/c$
Unit cell dimensions	a = 10.0722(1) Å<br>b = 15.3500(1) Å<br>c = 11.0263(1) Å<br>α = 90°<br>β = 92.721(10)°<br>γ = 90°	a = 9.2256(2) Å<br>b = 9 5320(2) Å<br>c = 22.5042 (5) Å<br>α = 95.965(2)°<br>β = 96.641(2)°<br>γ = 114.486(2)°	a = 6.2987(6) Å<br>b = 10.0280(9) Å<br>c = 11.3301(12) Å<br>α = 72.341(9)°<br>β = 78.427(8)°<br>γ = 72.315(8)°	a = 9.7948(1) Å<br>b = 10.0560(1) Å<br>c = 16.5671(1) Å<br>α = 82.023(1)°<br>β = 77.240(1)°<br>γ = 74.474(1)°	a = 9.5897(3)Å<br>b = 30.6431(8) Å<br>c = 11.1601(3)Å<br>α = 90°<br>β = 101.287(2)°<br>γ = 90°
Volume	1702.84(3) Å^3	1763.49(8) Å^3	645.04(12) Å^3	1527.91(3) Å^3	3216.06(16) Å^3
Z	4	4	1	2	4
Density (calculated)	1.047 g/cm^3	1.026 g/cm^3	1.503 g/cm^3	1.296 g/cm^3	1.298 g/cm^3
Absorption coefficient	0.507 mm^{-1}	0.538 mm^{-1}	5.445 mm^{-1}	2.593 mm^{-1}	1.941 mm^{-1}
F(000)	600	608	300	628	1320
Crystal size (mm^3)	0.408 x 0.131 x 0.126	0.4 x 0.27 x 0.2	0.112 x 0.037 x 0.023	0.221 x 0.185 x 0.122	0.203 x 0.08 x 0.054
Crystal color, habit	colorless needle	colorless block	orange, block	red, block	red, needle
Theta range for data collection	4.395 to 73.326 °	4.012 to 71.657 °	4.124 to 63.610°	4.580 to 80.339°	2.265 to 33.096°
Index ranges	$-12 \le h \le 12$, $-19 \le k \le 18$, $-13 \le l \le 9$	$-11 \le h \le 11$, $-11 \le k \le 11$, $-27 \le l \le 27$	$-7 \le h \le 7$, $-11 \le k \le 11$, $-13 \le l \le 13$	$-12 \le h \le 12$, $-10 \le k \le 12$, $-21 \le l \le 20$	$-13 \le h \le 14$, $-46 \le k \le 12$, $-16 \le l \le 17$
Reflections collected	16 703	24 293	3848	39 331	79257
Independent reflections	3380 [R(int) = 0.0225]	6780 [R(int) = 0.0236]	3848	6567 [R(int) = 0.0373]	10969 [R(int) = 0.0509]
Absorption correction	Gaussian	Gaussian	Gaussian	Multi-scan	Multi-scan
Max. and min. transmission	1.000 and 0.676	1.000 and 0.581	0.924 to 0.651	1.000 and 0.897	1.000 and 0.688
Refinement method	Full-matrix least-squares on F^2	Full-matrix least-squares on F^2	Full-matrix least-squares on F^2	Full-matrix least-squares on F^2	Full-matrix least-squares on F^2
Data / restraints / parameters	3380 / 0 / 181	6780 / 0 / 363	3848 / 0 / 134	6567 / 0 / 349	10969 / 0 / 349
Goodness-of-fit on F^2	1.073	1.044	1.009	1.038	1.015
Final R indices [I>2sigma(I)]	R_1 = 0.0362, wR_2 = 0.0922	R_1 = 0.0451, wR_2 = 0.1174	R_1 = 0.0787, wR_2 = 0.2034	R_1 = 0.0276, wR_2 = 0.0681	R_1 = 0.0381, wR_2 = 0.0756
R indices (all data)	R_1 = 0.0381, wR_2 = 0.0937	R_1 = 0.0499, wR_2 = 0.1213	R_1 = 0.0866, wR_2 = 0.2093	R_1 = 0.0282, wR_2 = 0.0685	R_1 = 0.0581, wR_2 = 0.0807
Largest diff. peak and hole	0.255 and -0.222 e/ Å^{-3}	0.352 and -0.192 e/ Å^{-3}	1.93 and -1.29 e Å^{-3}	0.337 and -0.572 e Å^{-3}	0.849 and -0.492 e Å^{-3}

Table S3: Crystal Data and Summary of X–ray Data Collection (cont.)

	***meso*-{A[2t]}$_2$ (7)**
Empirical formula	$C_{22}H_{42}$
Formula weight	306.58
Temperature	294.0(2) K
Wavelength	1.54184 Å
Crystal system	Triclinic
Space group	*P*-1
Unit cell dimensions	*a* = 6.1994(4) Å
	b = 9.0864(5) Å
	c = 9.6578(4) Å
	α = 91.363(4)°
	β = 90.556(4)°
	γ = 94.247(5)°
Volume	542.34(5) Å^3
Z	1
Density (calculated)	0.939 g/cm^3
Absorption coefficient	0.370 mm^{-1}
F(000)	174
Crystal size (mm^3)	0.186 x 0.144 x 0.035
Crystal color, habit	colorless, plate
Theta range for data collection	4.591 to 75.643°
Index ranges	$-7 \leq h \leq 7$, $-11 \leq k \leq 11$, $-12 \leq l \leq 10$
Reflections collected	2126
Independent reflections	5356 [*R*(int) = 0.0382]
Absorption correction	Multi-scan
Max. and min. transmission	1.000 and 0.853
Refinement method	Full-matrix least-squares on F^2
Data / restraints / parameters	2126 / 0 / 106
Goodness-of-fit on F^2	1.068
Final R indices [*I*>2sigma(I)]	R_1 = 0.0621, wR_2 = 0.1876
R indices (all data)	R_1 = 0.0806, wR_2 = 0.2043
Largest diff. peak and hole	0.250 and -0.161 e Å^{-3}
